# Effect of Genetic Variants of Gonadotropins and Their Receptors on Ovarian Stimulation Outcomes: A Delphi Consensus

**DOI:** 10.3389/fendo.2021.797365

**Published:** 2022-02-01

**Authors:** Alessandro Conforti, Frank Tüttelmann, Carlo Alviggi, Hermann M. Behre, Robert Fischer, Liang Hu, Nikolaos P. Polyzos, Dana Chuderland, Gottumukkala Achyuta Rama Raju, Thomas D’Hooghe, Manuela Simoni, Sesh K. Sunkara, Salvatore Longobardi

**Affiliations:** ^1^ Department of Neuroscience, Reproductive Science and Odontostomatology, University Federico II, Naples, Italy; ^2^ Institute of Reproductive Genetics, University of Münster, Münster, Germany; ^3^ Center for Reproductive Medicine and Andrology, University Hospital Halle, Martin Luther University Halle-Wittenberg, Halle, Germany; ^4^ Department of Gynecological Endocrinology and Reproductive Medicine, Fertility Center Hamburg, Hamburg, Germany; ^5^ Clinical Research Center for Reproduction and Genetics in Hunan Province, Reproductive and Genetic Hospital of CITIC-XIANGYA, Changsha, China; ^6^ Institute of Reproductive and Stem Cell Engineering, School of Basic Medical Science, Central South University, Changsha, China; ^7^ Department of Obstetrics, Gynecology and Reproductive Medicine, Dexeus University Hospital, Barcelona, Spain; ^8^ Global Medical Affairs Fertility, Merck Healthcare KGaA, Darmstadt, Germany; ^9^ Division of Gynaecology, Krishna IVF Clinic, Visakhapatnam, Andhra Pradesh, India; ^10^ Research Group Reproductive Medicine, Department of Development and Regeneration, Organ Systems, Group Biomedical Sciences, KU Leuven (University of Leuven), Leuven, Belgium; ^11^ Department of Obstetrics, Gynecology, and Reproductive Sciences, Yale School of Medicine, New Haven, CT, United States; ^12^ Unit of Endocrinology, Department of Biomedical, Metabolic and Neural Sciences, University of Modena and Reggio Emilia, Modena, Italy; ^13^ Faculty of Life Sciences and Medicine, King’s College London, London, United Kingdom; ^14^ Global Clinical Development, Research and Development, Merck KGaA, Darmstadt, Germany

**Keywords:** single nucleotide polymorphisms (SNPs), genetics, genetic variants, gonadotropins, FSHB/FSHR, LHB/LHCGR, ovarian stimulation and response, assisted reproductive technology (ART)

## Abstract

**Background:**

A Delphi consensus was conducted to evaluate the influence of single nucleotide polymorphisms (SNPs) in genes encoding gonadotropin and gonadotropin receptors on clinical ovarian stimulation outcomes following assisted reproductive technology (ART) treatment.

**Methods:**

Nine experts plus two Scientific Coordinators discussed and amended statements plus supporting references proposed by the Scientific Coordinators. The statements were distributed *via* an online survey to 36 experts, who voted on their level of agreement or disagreement with each statement. Consensus was reached if the proportion of participants agreeing or disagreeing with a statement was >66%.

**Results:**

Eleven statements were developed, of which two statements were merged. Overall, eight statements achieved consensus and two statements did not achieve consensus. The statements reaching consensus are summarized here. (1) SNP in the follicle stimulating hormone receptor (*FSHR*), rs6166 (c.2039A>G, p.Asn680Ser) (N=5 statements): Ser/Ser carriers have higher basal FSH levels than Asn/Asn carriers. Ser/Ser carriers require higher amounts of gonadotropin during ovarian stimulation than Asn/Asn carriers. Ser/Ser carriers produce fewer oocytes during ovarian stimulation than Asn/Asn or Asn/Ser carriers. There is mixed evidence supporting an association between this variant and ovarian hyperstimulation syndrome. (2) SNP of *FSHR*, rs6165 (c.919G>A, p.Thr307Ala) (N=1 statement): Few studies suggest Thr/Thr carriers require a shorter duration of gonadotropin stimulation than Thr/Ala or Ala/Ala carriers. (3) SNP of *FSHR*, rs1394205 (−29G>A) (N=1 statement): Limited data in specific ethnic groups suggest that A/A allele carriers may require higher amounts of gonadotropin during ovarian stimulation and produce fewer oocytes than G/G carriers. (4) SNP of FSH β-chain (*FSHB*), rs10835638 (−211G>T) (N=1 statement): There is contradictory evidence supporting an association between this variant and basal FSH levels or oocyte number. (5) SNPs of luteinizing hormone β-chain (*LHB*) and LH/choriogonadotropin receptor (*LHCGR*) genes (N=1 statement): these may influence ovarian stimulation outcomes and could represent potential future targets for pharmacogenomic research in ART, although data are still very limited.

**Conclusions:**

This Delphi consensus provides clinical perspectives from a diverse international group of experts. The consensus supports a link between some variants in gonadotropin/gonadotropin receptor genes and ovarian stimulation outcomes; however, further research is needed to clarify these findings.

## Introduction

Infertility is a significant global health problem and socioeconomic burden, affecting 15% of childbearing-age individuals worldwide and with an increasing prevalence over the last two decades (1). Assisted reproduction technology (ART) has provided a critical tool for addressing reproductive challenges in men and women (2–5). However, ART remains an area with unmet clinical needs. According to the International Committee for Monitoring Assisted Reproductive Technology (ICMART), the global *in vitro* fertilization (IVF)/intracytoplasmic sperm injection (ICSI) combined delivery rates per fresh aspiration and frozen embryo transfer cycles in 2013 were 24.2% and 22.8%, respectively, with a cumulative delivery rate per aspiration of 30.4% (6).

Delivery rate is closely associated with the number of oocytes retrieved during ovarian stimulation (7). This relationship is even more evident considering how frozen cycles contribute to cumulative live birth rate (8). In this sense, the goal is to safely retrieve the highest number of mature oocytes in order to get the highest percentage of delivery rate per initiated ovarian stimulation cycle for ART treatment.

A critical step of ART is ovarian stimulation using gonadotropins, the aim of which is to obtain an optimum number of mature oocytes without the risk of ovarian hyperstimulation syndrome (OHSS) (9). Responses to gonadotropin stimulation are highly variable and dependent on individual patient factors. The prediction of ovarian response is critical to enable optimal and individualized management of ovarian stimulation. Current ovarian stimulation protocols use several parameters to predict ovarian response and optimize the dose of gonadotropins accordingly, including age, body mass index, ovarian reserve tests such as anti-Müllerian hormone (AMH), antral follicular count (AFC), endocrine status and baseline serum follicle stimulating hormone (FSH) (10–12). In particular, AMH and AFC are widely considered the best predictors of ovarian potential (13–16). However, ovarian reserve cannot fully explain the individual response to ovarian stimulation. For instance, a subgroup of women with normal ovarian reserve but suboptimal or poor response (hypo-responders) have been described (17–19). These patients have an “unexpected” reduced response to ovarian stimulation and are characterized by low prognosis to ART (17, 20, 21). The mechanisms underlying this unexpected ovarian resistance to ovarian stimulation are not fully understood, but it is believed that an individual’s genetics play a significant role (17, 22).

Several gene association studies have identified specific single nucleotide polymorphisms [SNPs (**Box 1**)] of gonadotropins and their receptors that could influence ovarian response (26–28). These include SNPs of the FSH receptor (*FSHR*) gene, FSH β-chain (*FSHB*) gene, luteinizing hormone β-chain (*LHB*) gene and *LH*/choriogonadotropin receptor (*LHCGR*) gene (**Table 1**). The identification of genetic variants that are able to predict ovarian response could pave the way to tailor ovarian stimulation on the basis of individual genotype profile. Unfortunately, these data are still controversial and, at times, contradictory or limited.

Box 1 **Single Nucleotide Polymorphisms (SNPs).**

- SNPs can occur in both non-coding and coding sequences of genes.
- SNPs in non-coding regions appear to affect transcription as well as non-coding RNAs that can also influence gene expression (23).
- SNPs in coding regions can be synonymous or nonsynonymous.
  - Synonymous SNPs do not produce altered coding sequences, since some amino acids are coded for by more than one three-base-pair codon. Although these are often referred to as ‘silent’ polymorphisms, they can still affect the function of a gene (24).
  - Nonsynonymous SNPs can be missense (a single change of nucleotide base resulting in a change in amino acid) or nonsense (a point mutation in a sequence of DNA that results in premature termination of protein synthesis and in a truncated and usually non-functional protein product (25).
- Specific SNPs are catalogued by researchers and databases according to a non-redundant accession number, the reference SNP cluster identifier (rs).

**Table 1:** SNPs identified in gonadotropin isoforms and gonadotropin receptors.

SNP	Gene	Nucleotide change	Amino acid substitution	Molecular characteristics: Homozygous *vs* heterozygous states	Clinical relevance: Homozygous *vs* heterozygous states
rs6165*	*FSHR*	c.919G>A	p.Thr307Ala	Amino acid substitution results in a change from a polar to a nonpolar, hydrophobic amino acid and removal of a potential O-linked glycosylation site (29, 30).	Higher number of retrieved oocytes (31) and MII oocytes (32), and shorter duration of ovarian stimulation in Ala/Ala carriers versus Thr/Thr or Ala/Thr carriers (31).
rs6166*	*FSHR*	c.2039A>G	p.Asn680Ser	Amino acid substitution results in a potential phosphorylation site in the intracellular domain of the receptor (29, 30).	Higher basal gonadotropin levels, higher r-hFSH consumption, decreased estradiol levels on the day of hCG administration, lower number of retrieved oocytes and a higher incidence of hypo-responders in Ser/Ser versus Asn/Asn carriers (33–37). Asn/Asn carriers at an increased risk of OHSS versus Ser/Ser carriers (38).
rs1394205	*FSHR*	−29G>A	N/A	SNP in the promoter region of the *FSHR*, upstream of the translational initiation codon (39).	Reduced expression of *FSHR* and lower basal FSH levels in AA homozygotes versus GG homozygotes or AG heterozygotes (31, 39).
rs2293275	*LHCGR*	c.935A>G	p.Asn312Ser	Amino acid substitution is located near a glycosylation site (40)	Higher r-hFSH and r-hLH consumption and reduced sensitivity to LH with Ser/Ser carriers versus Asn/Asn carriers (41, 42). Significantly higher live birth rate with Ser/Ser carriers versus Asn/Asn or Asn/Ser carriers (p=0.043) (40).
rs1800447 (*v-betaLH*)	*LHB*	c.82T>C	p.Trp8Arg	v-betaLH amino acid substitution results in an extra glycosylation site in the β subunit, leading to a second oligosaccharide side-chain to Asn13. The *v-betaLH* homozygous variant has an elevated bioactivity *in vitro* but with a significantly shorter half-life versus wild type *LHB* (43).	Characterized by a less active form of LH that does not adequately support FSH activity during ovarian stimulation (43).
rs34349826 (*v-betaLH*)	*LHB*	c.104A>G	p.Ile35Thr		Associated with elevated testosterone levels in women withPCOS (44).
rs10835638	*FSHB*	211G>T	N/A	SNP in the promoter of *FSHB* influences gene transcription (30)	Poor response to ovarian stimulation, higher basal FSH/LH levels, lower AFC, and lower retrieved numbers of oocytes, MII oocytes and embryos with GT heterozygotes versus GG wild type (45–47)

*rs6165 and rs6166 are in linkage disequilibrium, apart from in some African populations (30, 31). Linkage disequilibrium is defined as the non-random association of alleles of different loci that are inherited co-ordinately (**Box 2**).

A, adenine; AFC, antral follicle count; Ala, alanine; Arg, arginine; Asn, asparagine; c., coding DNA sequence; C, cytosine; FSH, follicle-stimulating hormone; FSHB, follicle-stimulating hormone beta subunit; FSHR, follicle-stimulating hormone receptor; G, guanine; Ile, isoleucine; LH, luteinizing hormone LHB, luteinizing hormone β-chain; LHCGR, luteinizing hormone/choriogonadotropin receptor; MII, metaphasis II; N/A, not applicable; p., protein-level amino acid sequence; PCOS, polycystic ovary syndrome; r-hLH, recombinant human luteinizing hormone; r-hFSH recombinant human follicle-stimulating hormone; Ser, serine; SNP, single nucleotide polymorphism; T, thymine; Trp, tryptophan; Thr, threonine.

The aim of the current Delphi Consensus study was to generate a series of literature-supported consensus statements regarding the most relevant genetic variants of gonadotropin and gonadotropin receptors involved in ovarian response.

## Assessment of Statements According to Delphi Consensus Process

### Role of the Sponsor

The Delphi consensus was coordinated by a healthcare consulting and training company (Sanitanova Srl, Milan, Italy). The consensus concept was initiated and funded by Merck KGaA, Darmstadt, Germany. The sponsor was involved early in the process, defining the overarching topic to be discussed, but did not participate in the development of the statements or in any of the meetings or discussions involved in developing the Delphi consensus. The statements were, therefore, developed independently of the industry sponsor. The authors from Merck KGaA, Darmstadt, Germany, were only involved in the development of the manuscript, critically revising it for important intellectual content, especially in the Introduction, Results and Discussion sections, but could not alter the consensus statements in any way.

### Consensus Participants

The Delphi consensus involved a Scientific Board, comprising two Scientific Coordinators (AC and FT) and nine additional experts (**Table 2**). Scientific Board members were selected based on their recognized expertise in Reproductive Genetics proven by literature contributions in this field (**Supplementary Table 1**). Our goal was to have diverse coverage involving experienced panel members from across the world. Each member of the Scientific Board suggested an additional two or three experts, resulting in a panel of 36 experts (the Extended Panel), which comprised nine members of the Scientific Board (excluding the two Scientific Coordinators) plus 27 additional experts. Written informed consent was obtained from all Consensus participants for the publication of their name in **Table 2**.

**Table 2:** Participants involved in rounds 1–3 of the consensus.

Name	Country	Round 1 (Web conference*)		Round 2 (Online survey)	Round 3 (Web conference*)	
		06 October 2020 (09:00 CET)	08 October 2020 (16:30 CET)	10 November to 01 December 2020	02 December 2020 (09:00, CET)	14 December 2020 (16:30, CET)
**Scientific Coordinators**						
Alessandro Conforti	Italy	X	X		X	X
Frank Tüttelmann	Germany	X	X		X	X
**Scientific Board**						
Carlo Alviggi	Italy		X	X		X
Hermann M. Behre	Germany		X	X		X
Robert Fischer	Germany		X	X		X
José Gonçalves Franco Junior	Brazil	X		X		X
Liang Hu	China	X		X	X	
Nikolaos P. Polyzos	Spain	X		X	X	
Gottumukkala Achyuta Rama Raju	India	X		X	X	
Manuela Simoni	Italy	X		X	X	
Sesh K. Sunkara	UK		X	X		X
**Extended Panel**						
Claus Yding Andersen	Denmark			X		
Rafael Bernabeu	Spain			X		
Bianca Bianco	Brazil			X		
Peter Humaidan	Denmark			X		
Kim Jonas	UK			X		
João Sabino Lahorgue da Cunha Filho	Brazil			X		
Dimitris Loutradis	Greece			X		
Joop Se Laven	Netherlands			X		
Dolors Manau	Spain			X		
Ana Neves	Portugal			X		
Caio Parente Barbosa	Brazil			X		
Matheus Roque	Brazil			X		
Ippokratis Sarris	UK			X		
Laura Vagnini	Brazil			X		
Daniele Santi	Italy			X		
Antonio La Marca	Italy			X		

*Due to different time zones one web conference was held in the morning and a second in the afternoon.

Written informed consent was obtained from all Consensus participants for the publication of their name in **Table 2**.

### The Consensus Process

**Figure 1:**
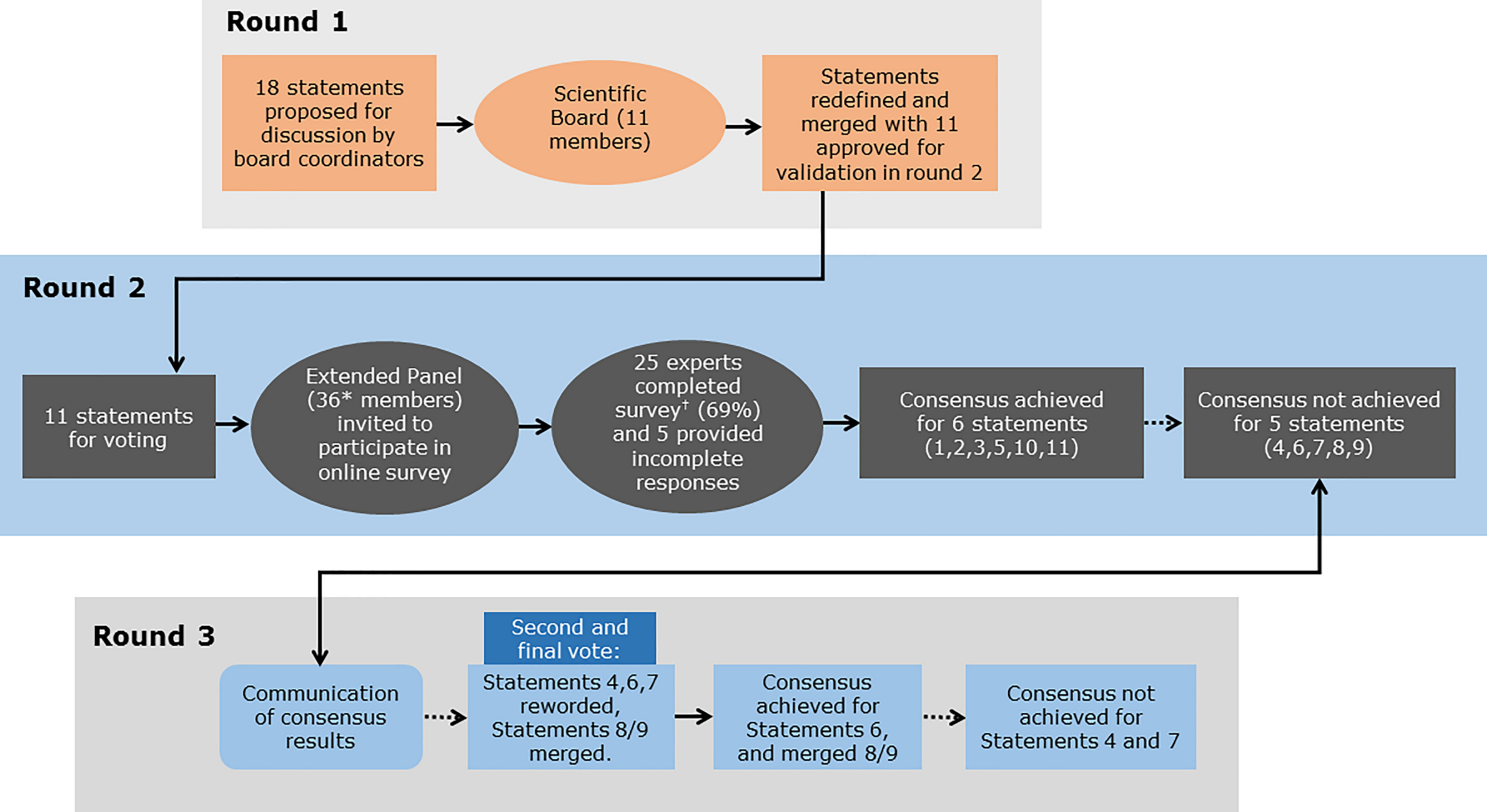
Overview of the Delphi consensus process and outcomes. Round 1: Statements and supporting references initially developed by the two Scientific Coordinators were discussed and amended by the 11 members of the Scientific Board. Round 2: An Extended Panel of 36 experts were invited to vote on their level of agreement or disagreement with each statement in an online survey, of which 25 experts completed the survey and five provided incomplete survey responses. Round 3: The consensus results were communicated to the participating experts. Statements that did not achieve consensus in Round 2 were discussed and revised or merged. The Extended Panel then voted on their level of agreement or disagreement with the revised statements. *9 members of the Scientific Board and 27 additional experts suggested by the Scientific Board; ^†^9 members of the Scientific Board and 16 additional experts suggested by the Scientific Board.

The Delphi consensus comprised three rounds (**Figure 1**). During Round 1, statements and supporting references initially developed by the two Scientific Coordinators were discussed and amended by the 11 members of the Scientific Board during two web conferences (**Table 2**). The statements and references to be used in Round 2 were approved by the Scientific Board. During Round 2, an online survey was conducted, in which the Extended Panel of 36 experts were invited to vote anonymously on their level of agreement or disagreement with the statements approved by the Scientific Board in Round 1. Voting was conducted using a five-point Likert-type scale (1=Absolutely agree; 2=Agree; 3=Neither agree nor disagree; 4=Disagree; 5=Absolutely disagree). Participants were also asked to provide the main reason(s) for their response in an open-ended response field. Of the 36 invited experts, 25 completed the survey (nine members of the Scientific Board [excluding the two Scientific Coordinators] plus 16 additional experts) and five provided incomplete survey responses. Consensus was considered to be achieved if the proportion of participants either agreeing with a statement (responding “agree” or “absolutely agree”) or disagreeing with a statement (responding “disagree” or “absolutely disagree”) exceeded 66% (48, 49). During Round 3, the consensus results were communicated to the participating experts *via* two web conferences (**Table 2**). Statements that did not achieve consensus in Round 2 were discussed, revised and/or merged by the scientific board. The newly reworded statements were shared with the Extended Panel for voting on their level of agreement or disagreement through an online survey during Round 3.

## Results of the Consensus and Actionable Recommendations (Including Supportive Evidence)

### Overall Results

A total of 18 statements with supporting references were proposed by the Scientific Coordinators. Following discussion and refinement of these statements by the Scientific Board during Round 1, a total of 11 statements with supporting references were approved by the Scientific Board and included in the online survey in Round 2 (**Table 3**). The Extended Panel who participated in the online survey comprised fertility experts from a number of different regions, including Europe, Asia, and South America.

**Table 3:** Statements for online voting in rounds 2 and 3.

Statement	
**Statements related to SNP of *FSHR*: rs6166, c.2039 A>G, p.Asn680Ser**	
**1.**	Ser/Ser carriers of *FSHR* (*FSHR* rs6166, c.2039A>G, p.Asn680Ser) require higher amounts of gonadotropin during ovarian stimulation than Asn/Asn carriers
**2.**	Ser/Ser carriers of *FSHR* (*FSHR* rs6166, c.2039A>G, p.Asn680Ser) showed higher basal levels of FSH compared with Asn/Asn carriers
**3.**	Ser/Ser carriers of *FSHR* (*FSHR* rs6166, c.2039A>G, p.Asn680Ser) produce fewer oocytes in response to ovarian stimulation than Asn/Asn and Asn/Ser carriers
**4.**	Ser/Ser carriers of *FSHR* (*FSHR* rs6166, c.2039A>G, p.Asn680Ser) produce fewer metaphase II oocytes after ovarian stimulation than Asn/Asn and Asn/Ser carriers
4. (revote*)	Available data in the literature suggest that Ser/Ser carriers of *FSHR* (*FSHR* rs6166, c.2039A>G, p.Asn680Ser) tend to produce fewer metaphase II oocytes after ovarian stimulation than Asn/Asn and Asn/Ser carriers
**5.**	There is mixed evidence supporting an association between the *FSHR* Asn680Ser variant (*FSHR* rs6166, c.2039A>G, p.Asn680Ser) and ovarian hyperstimulation syndrome
**Statements related to SNP of *FSHR*: rs6165, c.919G>A, p.Thr307Ala**	
**6.**	Thr/Thr carriers of *FSHR* (*FSHR* rs6165, c.919G>A, p.Thr307Ala) require a shorter duration of gonadotropin stimulation than Thr/Ala carriers^†^
6. (revote*)	Few studies suggest that Thr/Thr carriers of *FSHR* (*FSHR* rs6165, c.919G>A, p.Thr307Ala) require a shorter duration of gonadotropin stimulation than Thr/Ala and Ala/Ala carriers
**7.**	Thr/Thr carriers of *FSHR* (*FSHR* rs6165, c.919G>A, p.Thr307Ala) produce a higher number of oocytes in response to ovarian stimulation than Thr/Ala and Ala/Ala carriers^†^
7. (revote*)	Ala/Ala carriers of *FSHR* (*FSHR* rs6165, c.919G>A, p.Thr307Ala), which is in strong linkage disequilibrium with the Ser/Ser *FSHR* variant (*FSHR* rs6166, c.2039A>G, p.Asn680Ser), produce fewer oocytes in response to ovarian stimulation than Thr/Ala and Thr/Thr carriers^†^
**Statements related to SNP of *FSHR*: rs1394205, −29G>A**	
**8.**	Limited data suggest that A/A allele carriers of the *FSHR* −29 variant (rs1394205, −29G>A) require higher amounts of gonadotropin during ovarian stimulation than G/G carriers
**9.**	Limited data suggest that A/A allele carriers of the *FSHR* −29 variant (rs1394205, –29G>A) produce fewer oocytes in response to ovarian stimulation than GG carriers
8/9 merged (revote*)	Limited data in specific ethnic groups suggest that A/A allele carriers of the *FSHR* −29 variant (rs1394205, −29G>A) require higher amounts of gonadotropin and produce fewer oocytes in response to ovarian stimulation than G/G carriers
**Statements related to SNP of *FSHB*: rs10835638, −211G>T**	
**10.**	The data on an association of *FSHB* (*FSHB* rs10835638, −211G>T) with basal levels of FSH and production of oocytes in response to ovarian stimulation are contradictory
**Statements related to SNPs of the *LHB/LHCGR* genes**	
**11.**	Limited data suggest that polymorphisms of the *LHB/LHCGR* genes (*V-betaLH* rs1800447, c.82T>C, p.Trp8Arg; *V-betaLH* rs34349826, c.104 A>G, p.Ile35Thr; *LHCGR* rs2293275, c.935A>G, p.Asn312Ser) can influence ovarian stimulation outcomes and may represent targets for pharmacogenomic research in ART

*Any statement that did not achieve consensus in Round 2 was discussed and reworded in Round 3, in order to revote with the Extended Panel. ^†^FSHR rs6166 (c.2039A>G, p.Asn680Ser) and FSHR rs6165 (c.919G>A, p.Thr307Ala) are in linkage disequilibrium, except in some African populations.

A, adenine; Ala, alanine; Asn, asparagine; Arg, arginine; ART, assisted reproductive technology; c., coding DNA sequence; C, cytosine; FSH, follicle-stimulating hormone; FSHB, follicle-stimulating hormone β-chain; FSHR, follicle-stimulating hormone receptor; G, guanine; Ile, isoleucine; LH, luteinizing hormone; LHB, luteinizing hormone β-chain; LHCGR, luteinizing hormone/choriogonadotropin receptor; SNP, single nucleotide polymorphism; T, thymine; Trp, tryptophan; Thr, threonine.

No statements achieved 100% agreement. Consensus was achieved for six statements (Statements 1, 2, 3, 5, 10 and 11). A high level of agreement (≥80% of votes were ‘agree’ or ‘absolutely agree’) was achieved for three statements (Statements 1, 10 and 11). Five statements failed to reach consensus (Statements 4, 6, 7, 8, and 9) and were discussed and amended during Round 3. Statements 4 and 7, which had a total agreement level of 50% (i.e. only 50% of votes were ‘agree’ or ‘absolutely agree’) and 52%, respectively, after the first vote, were reworded but failed to reach consensus after a second round of voting (57% and 62% total agreement, respectively). Statement 6, which had a total agreement level of 55% after the first vote, was reworded and reached consensus after a second vote (67% total agreement). Statements 8 and 9, which both had a total agreement level of 50% after the first vote, were merged and reworded, and achieved consensus after a second vote (76% total agreement). For two statements (Statements 5 and 7 [revote]) one expert voted ‘absolutely disagree’. The reasons that experts provided when voting to disagree with a given statement are shown in **Supplementary Table 2**.

## Statements Related to SNP of *FSHR*: rs6166, c.2039A>G, p.Asn680Ser

### Statement 1: Ser/Ser Carriers of *FSHR* (*FSHR* rs6166, c.2039A>G, p.Asn680Ser) Require Higher Amounts of Gonadotropin During Ovarian Stimulation than Asn/Asn Carriers

**Figure 2:**
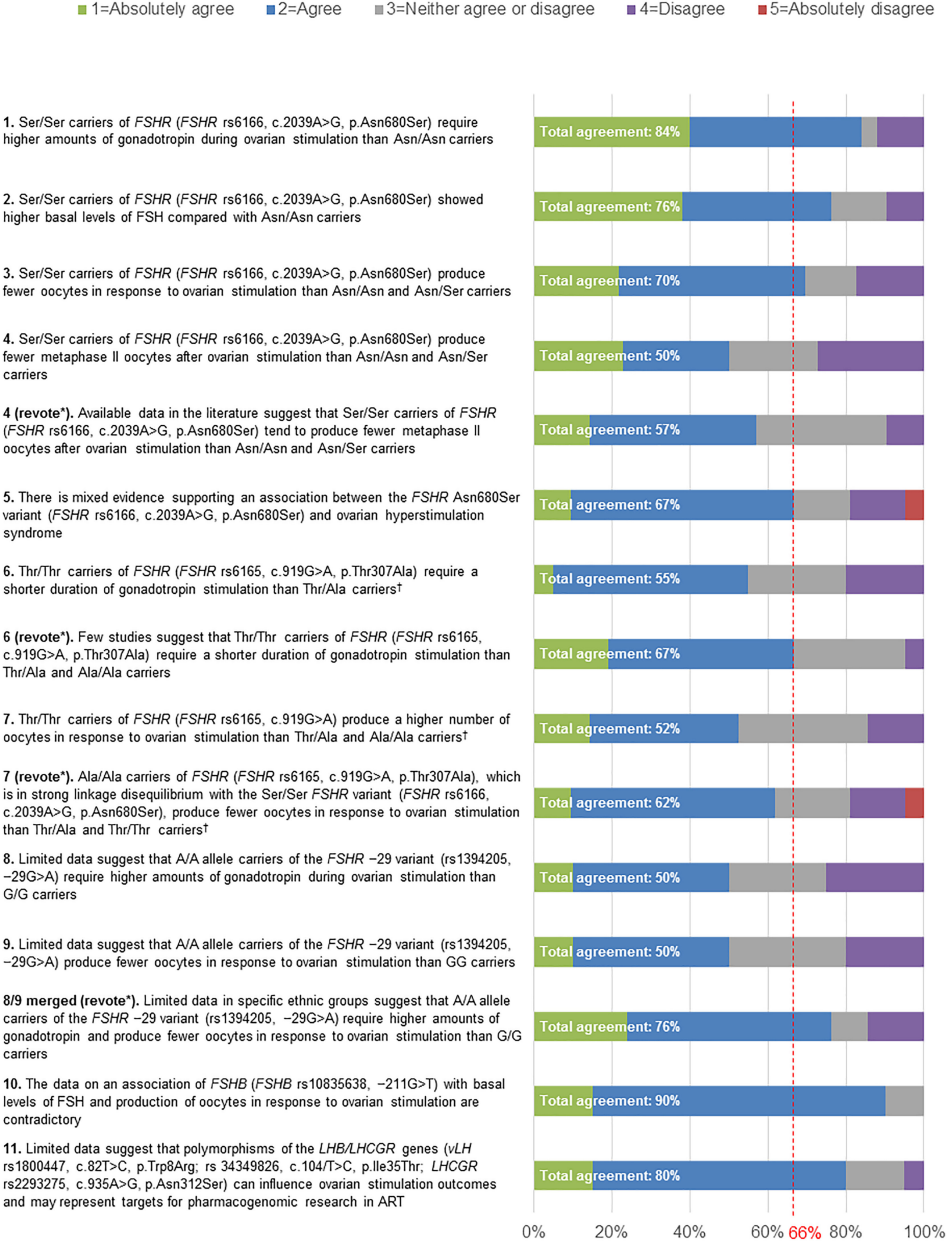
Level of agreement/disagreement with each statement (Rounds 2 and 3). The Extended Panel voted on their level of agreement or disagreement with each of the 11 statements using a 5-point Likert scale (1=Absolutely agree; 2=Agree; 3=Neither agree nor disagree; 4=Disagree; 5=Absolutely disagree). Consensus was considered to have been achieved if the proportion of participants either agreeing with the statement (responding 1 or 2) or disagreeing with the statement (responding 4 or 5) exceeded 66%. *Any statement that did not achieve consensus in Round 2 was discussed and reworded in Round 3, in order to revote with the Extended Panel. ^†^ *FSHR* rs6166 (c.2039A>G, p.Asn680Ser) and *FSHR* rs6165 (c.919G>A, p.Thr307Ala) are in linkage disequilibrium, except in some African populations.

This statement received 84% agreement from the Extended Panel (**Figure 2**). The reasons provided by participants for disagreeing with this statement included the absence of data from RCTs of an adequate size and the fact that a meta-analysis failed to show significant differences in gonadotropin consumption. Furthermore, one expert suggested that the extent to which *FSHR* rs6166 affected gonadotropin consumption depended on the study population (**Supplementary Table 2**).

A number of studies support a role for the *FSHR* rs6166 (c.2039A>G, p.Asn680Ser) variant as a prognostic indicator of ovarian response to FSH stimulation (33–37). The Ser/Ser variant was associated with higher basal levels of FSH (33, 34, 36), a higher total dose of gonadotropins required during ovarian stimulation (33, 34, 36), lower peak estradiol levels (34–36) and fewer retrieved oocytes (36). Collectively these studies suggest that the Ser/Ser variant is associated with a reduced sensitivity of the FSHR to exogenous FSH. A randomized controlled trial (RCT) demonstrated that this reduced sensitivity of the FSHR may be overcome by increasing the FSH dose (35).

In a study by Perez Mayorga et al. in 161 women aged <40 years undergoing ovarian stimulation in Germany, significantly more exogenous FSH was required to achieve ovulation stimulation and oocyte retrieval with the Ser/Ser variant compared with the Asn/Asn variant or the Asn/Ser variant (mean number of FSH ampoules [SEM]: 46.8 [5.0] *vs* 31.8 [2.4] *vs* 40.7 [2.3], respectively; p<0.01). Multiple linear regression analysis revealed that the number of ampoules of exogenous FSH could be predicted from both the type of polymorphism and basal FSH level (p<0.001) (33). Furthermore, a study in 522 Japanese women (mean age 31.8 years) reported that the distribution of *FSHR* rs6166 polymorphisms was 12.1% for the Ser/Ser variant compared with 41.0% for the Asn/Asn variant and 46.9% for the Asn/Ser variant (34). A higher dose of exogenous FSH was required to achieve ovulation stimulation in women with the Ser/Ser variant than women with the Asn/Ser variant (25%; p<0.05) or Asn/Asn variant (16%), although the latter comparison did not reach statistical significance.

In a study in 263 Korean women aged <40 years, Jun et al. reported significantly higher basal FSH levels in women with the Ser/Ser variant compared with those with the Asn/Asn or Asn/Ser variants (mean [SEM] 8.2 [0.9] IU/L *vs* 5.7 [0.3] IU/L and 6.0 [0.3] IU/L, respectively) (36). There was a trend towards lower peak estradiol levels and a higher exogenous FSH dose required for ovarian stimulation, but these did not reach statistical significance. The number of oocytes retrieved was lower in women with the Ser/Ser variant compared with those with the Asn/Asn or Asn/Ser variants (mean [SEM] 7.9 [0.8] *vs* 9.6 [0.6] and 10.2 [0.6], respectively). Furthermore, the clinical pregnancy rate was significantly lower in women with the Ser/Ser or Asn/Ser variants compared with those with the Asn/Asn variant (28.1% *vs* 31.1% *vs* 45.7%, respectively; p=0.013) (36). Similarly, in a study of 1250 Chinese women aged ≤38 years undergoing IVF/ICSI treatment (50), women with the Ser/Ser variant had higher basal FSH levels, required a higher dose of exogenous recombinant human FSH (r-hFSH) for ovarian stimulation and had fewer oocytes retrieved compared with women with the Asn/Asn or Asn/Ser variants. A logistic regression analysis demonstrated that the odds ratio (OR) of a poor ovarian response was 2.25 (95% CI 1.40, 3.58; p<0·001) for the Ser/Ser variant, compared with 1.79 (95% CI 1.28, 2.61; p<0.001) for the Asn/Ser variant (50).

In a prospective RCT of women aged <40 years undergoing ovarian stimulation for IVF/ICSI, carriers of the Ser/Ser variant were randomly assigned to receive hFSH (recombinant or urinary) 150 IU/day (n=24) or hFSH 225 IU/day (n=25), whereas carriers of the Asn/Asn variant (n=44) received hFSH 150 IU/day (35). Peak estradiol levels were significantly lower in the Ser/Ser variant carriers receiving hFSH 150 IU/day compared with the Asn/Asn variant carriers (mean [SEM] 5680 [675] pmol/L and 8679 [804] pmol/L, respectively; p=0.028); however, increasing the hFSH dose to 225 IU/day overcame the lower estradiol response in women with the Ser/Ser variant (7804 [983] pmol/L). This study suggests that the lower FSHR sensitivity associated with the Ser/Ser variant could be overcome by using higher FSH doses (35). In addition, a retrospective evaluation in 42 women aged <37 years compared genetic and clinical characteristics in those who required a higher cumulative dose of r-hFSH (>2500 IU; n=17) with those who required a lower cumulative dose of r-hFSH (<2500 IU; n=24) (37). The number of oocytes retrieved (p=0.0005) and embryos transferred (p=0.001) were significantly greater in women with a higher r-hFSH consumption compared with those with a lower r-hFSH consumption. The incidence of the Ser/Ser variant was higher in patients requiring a higher r-hFSH dose (p=0.02), suggesting that this genotype may be associated with a poor response to FSH, that may be overcome by higher FSH doses (37).

### Statement 2: Ser/Ser Carriers of *FSHR* (*FSHR* rs6166, c.2039A>G, p.Asn680Ser) Showed Higher Basal FSH Levels Compared With Asn/Asn Carriers

This statement received 76% agreement from the Extended Panel (**Figure 2**). The reasons given by participants for disagreeing with this statement included poor replication of data between studies, the fact that most studies were conducted in homogeneous populations and that the extent to which *FSHR* rs6166 affected basal FSH levels depended on the study population (**Supplementary Table 2**).

A number of studies have reported elevated basal FSH levels in Ser/Ser carriers of *FSHR* rs6166 (31, 33, 36, 46, 50–53). In a meta-analysis by Alviggi et al, Ser/Ser carriers had significantly higher basal FSH levels than Asn/Asn carriers (fixed random weighted mean difference [WMD] for Asn/Asn versus Ser/Ser −0.54 [95% CI −0.72, −0.36], p<0.00001, Bonferroni adjusted p<0.0001, *I^2 =^* 21%); based on data from one study (31). Furthermore, a study assessing serum FSH, LH and AMH levels in the follicular stage of the menstrual cycle in eumenorrheic healthy women without known fertility problems (n=169) and female partners of infertile couples (n=186) reported that the Ser/Ser variant was associated with significantly higher basal FSH levels compared with the Asn/Ser or Asn/Asn variants in the healthy group (G-allele affect [SE] 0.56 [0.19]; p=0.0046) (46).

A retrospective study in 1250 Chinese women with poor (≤5 oocytes) or good (>5 oocytes) ovarian response who were undergoing IVF/ICSI reported higher basal FSH levels in women with the Ser/Ser variant compared with the Asn/Asn or Asn/Ser variants (50). In another retrospective study in 136 women with either poor response (n=22), normal response (n=57) or high response (n=57), basal FSH levels (Day 2) were significantly higher in women with the Ser/Ser variant compared with the Asn/Ser or Asn/Asn variants (p<0.05) (53). Another retrospective study in 263 Korean women aged <40 years also reported significantly higher basal FSH levels in women with the Ser/Ser variant compared with the Asn/Asn or Asn/Ser variants (mean [SEM] 8.2 [0.9] IU/L *vs* 5.7 [0.3] IU/L and 6.0 [0.3] IU/L, respectively; p=0.001) (36).

Falconer et al. reported a higher distribution of the Ser/Ser variant compared with the Asn/Ser or Asn/Asn variants (41%, 24% and 35%, respectively) in 68 infertile women in Sweden (median age 33 [range 23–38] years). There was no significant difference in basal (Day 3) FSH levels between the Ser/Ser, Asn/Ser or Asn/Asn variants in a subpopulation of women with normal ovulatory reserve (mean [SE] 5.7 [1.7] *vs* 6.7 [1.3] *vs* 5.6 [1.9], respectively), although significantly higher FSH levels were detected at Day 10 in the women with the Ser/Ser variant compared with women with the Asn/Ser or Asn/Asn variants (mean [SE] 8.3 [2.8] *vs*. 6.3 [1.7] *vs* 6.9 [1.9], respectively; p<0.01). Additionally, FSH levels in women with the Ser/Ser variant at Day 3 of the cycle were significantly higher than at Day 10 (p<0.05) (52). *FSHR* genotypes were also evaluated in a cross-sectional study among 178 women (148 normogonadotropic anovulatory women, of whom 61 had polycystic ovary syndrome [PCOS], and 30 normo-ovulatory controls) (51). The Ser/Ser variant was significantly more prevalent in the anovulatory group compared with the normo-ovulatory control group (40% versus 16%). Furthermore, anovulatory women with the Ser/Ser variant had higher basal FSH serum levels (5.2 IU/L [range, 2.4–9.7 IU/L) than those with the Asn/Asn (4.6 IU/L [range, 1.4–5.8 IU/L) or Asn/Ser (4.5 IU/L [range, 1.8–9.7 IU/L) variants (p<0.01) (51). Finally, Perez Mayorga et al. assessed *FSHR* polymorphisms in 161 ovulatory women aged <40 years with couple-infertility attributed to male causes, tubal factor, or both (33). Distributions for the Ser/Ser, Asn/Asn and Asn/Ser variants were 26%, 29% and 45%, respectively. Basal FSH levels were significantly higher for women with the Ser/Ser variant compared with women with the Asn/Asn and Asn/Ser variants (p<0.05).

### Statement 3: Ser/Ser Carriers of *FSHR* (*FSHR* rs6166, c.2039A>G, p.Asn680Ser) Produce Fewer Oocytes in Response to Ovarian Stimulation than Asn/Asn and Asn/Ser Carriers

This statement received 70% agreement from the Extended Panel (**Figure 2**). The main reasons stated by participants for disagreeing with this statement included the fact that most data were from observational studies with a limited number of patients, and the absence of data from RCTs. Furthermore, one participant suggested that the extent to which *FSHR* rs6166 affected oocyte number was dependent on the study population (**Supplementary Table 2**).

A number of studies suggest that women with the *FSHR* rs6166 (c.2039A>G, p.Asn680Ser) Ser/Ser variant produce fewer oocytes in response to ovarian stimulation than women with the Asn/Asn or Asn/Ser variants, despite there being no statistically significant difference in the gonadotropin dose among women with the different polymorphisms (31, 36, 50, 54–59).

In a retrospective study in Mexican Mestizo women (n=224) there was a lower distribution for the Ser/Ser variant (9.8%) compared with the Asn/Asn variant (41.9%) or Asn/Ser variant (48.2%) (59). The Ser/Ser variant was associated with a significantly reduced number of retrieved oocytes following ovarian stimulation compared with the Asn/Asn or Asn/Ser variants (p<0.01) in normal oocyte donors. There was also a trend towards lower pregnancy rates in women with the Ser/Ser variant, which was stronger in a separate analysis of women with more Native American ancestry (OR 2.0 [95% CI 1.03, 3.90], p=0.04) (59). Furthermore, Alviggi et al. conducted a systematic review and meta-analysis of 33 studies, 21 of which (including 4425 women) reported the number of oocytes retrieved in relation to *FSHR* rs6166 (31). The number of oocytes retrieved was significantly lower in women with the Ser/Ser variant compared with women with the Asn/Asn variant (random WMD 0.84 [95% CI 0.19,1.49], p=0.01, Bonferroni adjusted p=0.03, *I^2^ = *76%), and was significantly lower in women with the Ser/Ser variant compared with the Asn/Ser variant (random WMD 0.88 [95% CI 0.12, 1.63], p=0.02, Bonferroni adjusted p=0.04, *I^2^ = *76%) (31). In addition, a cohort study in 1250 Chinese women aged ≤38 years undergoing IVF/ICSI treatment reported that women with the Ser/Ser variant had higher basal FSH levels, required a higher dose of exogenous gonadotropin for ovarian stimulation and had fewer oocytes retrieved compared with women carrying the Asn/Asn or Asn/Ser variants (50). A logistic regression analysis demonstrated that the OR of a poor ovarian response for women with the Ser/Ser variant was 2.25 (95% CI 1.40, 3.58; p<0.001) compared with the Asn/Asn variant (50).

A meta-analysis conducted by Tang et al. of 16 cohort studies (4278 women) determined, using a random effects model, that the number of oocytes retrieved in women with the Ser/Ser variant was significantly lower than in those with the Asn/Asn or Asn/Ser variants (WMD −1.36 [95% CI −1.85, −0.87] (58). Another meta-analysis of 11 studies (4020 women) demonstrated that women with the Ser/Ser variant were more likely to be poor responders (previously defined in other studies on this polymorphism as no more than 4–5 oocytes retrieved following ovarian stimulation) (50, 60) compared with the Asn/Asn or Asn/Ser variants (OR 1.61, p=0.08) (57). Furthermore, a prospective study in 450 Chinese women receiving ovarian stimulation for ART (56) reported higher basal levels of FSH in women with the Ser/Ser variant compared with the Asn/Asn or Asn/Ser variants (p<0.05), with numerically fewer oocytes retrieved in the women with the Ser/Ser variant compared with women with the Asn/Asn variant or the Asn/Ser variant (mean number of oocytes [SEM]: 11.12 [7.29] *vs* 13.07 [6.76] *vs* 13.20 [6.17], respectively). Moreover, the Ser/Ser variant was associated with an increased risk of poor response compared with the other two variants (p<0.05) (56).

A genotyping study that assessed the effect of genotype on ovarian responses in 300 women undergoing IVF/ICSI treatment, compared with a control group of 300 women with successful child birth, reported a reduction in the number of oocytes retrieved in women with the Ser/Ser variant compared with women with the Asn/Asn variant (p<0.02) (55). In addition, a study in 263 Korean women aged <40 years reported the retrieval of fewer oocytes in women with the Ser/Ser variant compared with the Asn/Asn or Asn/Ser variants (mean [SEM] 7.9 [0.8] *vs* 9.6 [0.6] vs 10.2 [0.6], respectively) (36). In another study, Loutradis et al. analysed polymorphisms in 125 women classified as ‘subfertile’ (n=79; defined as women who had previously undergone ovarian stimulation and who had a Day 3 FSH level of ≥9 IU/L [normal range 2–9 IU/L]) or ‘normo-ovulatory’ (n=46) (54). The distribution of the Ser/Ser, Asn/Ser and Asn/Asn variants were 45.5%, 22.7% and 31.8%, respectively, in the subfertile women. The number of oocytes retrieved for women with the Ser/Ser variant was significantly lower than those with the Asn/Ser variant (p<0.01) (54).

In a retrospective study in 170 women in Spain with conserved ovarian function undergoing ovarian stimulation (mean [SD] age 33 [2.55] years), the frequency of the Ser/Ser variant was higher in women with poor response (≤3 ovarian follicles) compared with the Ser/Asn or Asn/Asn variants (30% vs 13.9% vs 14.5% respectively; p=0.005). The number of oocytes retrieved for each variant was not reported in this study (61). Finally, in another retrospective study in Spain by the same author in 102 women undergoing ovarian stimulation (mean [SD] age 33 [2.55] years), 37% of women with poor ovarian response (≤3 ovarian follicles) had the Ser/Ser variant, compared with 21% of women with the Asn/Asn variant. However, in contrast to the other studies reported here, there was no difference in the number of oocytes retrieved between the Ser/Ser variant and the Asn/Asn or Asn/Ser variants (mean [range] number of oocytes: Ser/Ser: 5 [0–14] vs Asn/Asn + Asn/Ser: 5.3 [0–21]; p=0.85) (62). This may be due to the small sample size (102 women, of whom only 19 patients carried the Ser/Ser variant). Furthermore, the overall number of oocytes retrieved was low, regardless of variant (5.2 [range 0–21]), despite the fact that only 19% of patients were categorized as poor responders (62).

### Statement 4 (Revote): Available Data in the Literature Suggest that Ser/Ser Carriers of *FSHR* (*FSHR* rs6166, c.2039A>G, p.Asn680Ser) Tend to Produce Fewer Metaphase II Oocytes After Ovarian Stimulation Than Asn/Asn and Asn/Ser Carriers

This statement reached 50% agreement in the first round of voting (**Figure 2**). The wording of the statement was revised to reflect the fact that there are few large, prospective studies and no RCTs available regarding the influence of genotype on gonadotropin stimulation protocols. Furthermore, there were insufficient data on the comparison of homozygotic carriers of *FSHR* rs6166 (c.2039A>G, p.Asn680Ser) with grouped heterozygotic carriers. The reworded statement reached 57% agreement during re-voting (**Figure 2**); therefore, this statement did not achieve consensus. This statement received 10% disagreement from the Extended Panel, with 33% of experts neither agreeing nor disagreeing with the statement. The main motivation for disagreement was insufficient evidence to support this statement (**Supplementary Table 2**).

In a study including 455 consecutively enrolled women and 210 unselected women aged <40 years, the number of MII oocytes in a subgroup of women who underwent ICSI (n=317) was significantly lower in Ser/Ser carriers compared with Asn/Ser carriers or Asn/Asn carriers (mean [SD]: 6.1 [3.0] vs 7.1 [4.1] vs 8.2 [4.5]; unadjusted p=0.012; adjusted p=0.009 [adjusted for age]). (40). Furthermore, in a study of 104 prospectively enrolled women of Albanian ethnic population from the Kosovo Dukagjin region undergoing ICSI for male factor infertility, women with the Ser/Ser variant had a lower rate of MII oocytes (78.6%) compared with women with Asn/Asn (84.7%) or Asn/Ser (89.7%) variants (p=0.0258). In addition, there was a lower rate of immature MI oocytes progressing to MII state after 2-6 hours of *in vitro* incubation (5.6%) in women with the Ser/Ser variant compared with women with the Asn/Ser (11.6%) or Asn/Asn (8.4%) variants (p=0.0031) (65).

However, in contrast to these studies, a cross-sectional study of 384 women aged <40 years (mean age [SD]: 32.0 [3.82] years) undergoing IVF reported no significant difference in the number of mature oocytes retrieved in Ser/Ser variant carriers compared with Asn/Ser or Asn/Asn variant carriers (mean [SD]: 9.03 [5.6] vs 8.85 [5.3] vs 9.25 [6.0]). There was a trend towards a difference in the *a posteriori* enrolled validation cohort (n=233; mean [SD]: Ser/Ser 9.18 [5.2], Asn/Ser 10.5 [7.1], Asn/Asn 11.3 [7.1]) and the merged cohort (mean [SD]: Ser/Ser 9.05 [5.4], Asn/Ser 9.43 [6.1], Asn/Asn 10.1 [6.5]) (41). However, the differences between the variants were not significant in the study, validation or merged cohorts, suggesting no influence of the polymorphisms on receptor sensitivity for *in vitro* stimulation response (41).

A meta-analysis of five studies [n=1185 women (also including 41)] reported a numerically lower number of oocytes in women with the Ser/Ser variant compared with women with the Asn/Asn variant, although the difference was not significant after Bonferroni correction (fixed weighted mean difference 1.03, 95% CI 0.01 to 2.05; p=0.05. Bonferroni adjusted p=0.14; *I^2^ = *0%). No significant differences were observed between women with the Asn/Asn variant and those with the Asn/Ser variant (fixed weighted mean difference 0.79, 95% CI –0.05 to 1.62; *I^2^ = *0%) or between those with the Ser/Ser variant and those with the Asn/Ser variant (fixed weighted mean difference 0.34, 95% CI –0.57 to 1.26; *I^2 =^* 49%) (31).

### Statement 5: There Is Mixed Evidence Supporting an Association Between the *FSHR* Asn680Ser Variant (*FSHR* rs6166, c.2039A>G, p.Asn680Ser) and Ovarian Hyperstimulation Syndrome

This statement reached 67% agreement during the first round of voting (**Figure 2** **)**. The main reasons stated by participants for disagreeing with this statement included insufficient evidence and the fact that most data were from observational studies, with an absence of data from RCTs, or from studies in specific populations (**Supplementary Table 2**).

This statement was supported by evidence from three studies (38, 58, 66). A retrospective study of 586 women undergoing their first IVF treatment determined whether the *FSHR* rs6166 (c.2039A>G, p.Asn680Ser) predicted the likelihood of developing OHSS (38). In this study, 36 women (6%) developed OHSS, of whom none carried the Ser/Ser variant. *FSHR* rs6166 was associated with OHSS (*P*_trend_ = 0.004 and *P*_allele_ = 0.038), with carriers of Asn having an OR of 1.7 (95% CI 1.0, 2.8; p<0.04), compared with carriers of Ser. Women who developed OHSS were exposed to a lower total hormonal dose, yet produced more oocytes than those without OHSS (16 ± 8 vs 11 ± 6; p=0.001) (38). Conversely, a meta-analysis conducted by Tang et al., comprising 16 cohort studies and a total of 4287 women, reported no evidence for an association between *FSHR* rs6166 variants and OHSS (OR: 1.58, 95% CI: 0.41, 6.07) (58). Finally, in their retrospective study of 150 Indian women (n=50 in an assisted reproductive technology [ART] program and n=100 with proven fertility [control group]), Achrekar et al. explored the association between *FSHR* rs6166 variants and variable ovarian response, including the occurrence of OHSS (66). The distributions were 31%, 56%, and 13% in controls and 42%, 46%, and 12% in ART patients, for the Asn/Asn, Asn/Ser and Ser/Ser variants, respectively. (66). Patient age, basal FSH and LH levels, progesterone levels before and on the day of human choriogonadotropin (hCG) administration, number of pre-ovulatory follicles, number of oocytes retrieved, and pregnancy rates showed no statistically significant differences among groups, suggesting that treatment outcome was independent of the *FSHR* rs6166 variants. There were no statistically significant differences in any of the clinical parameters among women with the different variants, although women with the Ser/Ser variant showed higher serum estradiol levels before or on the day of hCG administration. OHSS developed in 50%, 26%, and 29% of women with the Ser/Ser, Asn/Ser, and Asn/Asn variants, respectively, although these values were not statistically significant (OR: 2.67) (66).

## Statements Related to SNP of *FSHR*: rs6165, c.919G>A, p.Thr307Ala

### Statement 6 (Revote): Few Studies Suggest That Thr/Thr Carriers of *FSHR* (*FSHR* rs6165, c.919G>A, p.Thr307Ala) Require a Shorter Duration of Gonadotropin Stimulation Than Thr/Ala and Ala/Ala Carriers

The original wording of this statement received 55% agreement during the first round of voting (**Figure 2**). Following discussion regarding the lack of prospective studies reporting on this outcome, the wording was revised and received 67% agreement after re-voting (**Figure 2**). The reasons given by participants for disagreeing with this statement included the fact that there was a limited number of studies and that the available studies had been poorly designed (**Supplementary Table 2**).

In one study including 450 Chinese women undergoing IVF due to male factor, tubal factor, or both, the length of stimulation was significantly different among women with different variants (although only a small absolute clinical difference was observed), with the longest duration of stimulation reported in women with the Ala/Ala variant: Thr/Thr 11.32 (2.15) days, Thr/Ala 12.02 (2.44) days and Ala/Ala 12.62 (2.92) days; p<0.05 (56). A prospective, cross-sectional study of 149 women in Brazil undergoing ART treatment due to male factor (n=93) or tubal factor (n=56) did not report on the stimulation length in women with different variants, but there was no significant difference in mean (SD) basal FSH levels (Thr/Thr 6.0 [2.0] versus Ala/Ala 6.41 [1.96] and Thr/Ala 6.49 [1.73], p=0.402), suggesting a comparable ovarian response for the alleles related to this variant (32).

In a meta-analysis of three studies (679 patients, including Yan 2013), a shorter duration of stimulation that approached statistical significance was reported between women with the Thr/Thr and Ala/Ala variants (random weighted mean difference −0.59 [95% CI −1.24, 0.05], *I^2^ = *60%, p=0.07). However, the duration of stimulation was significantly shorter in women with the Thr/Thr variants than in those with the Thr/Ala variant (fixed weighted mean difference −0.48 [95% CI −0.87, −0.10], p-0.01; Bonferroni adjusted p=0.04; *I^2^ = *44%), although there was no difference between women with the Ala/Ala variant and those with the Thr/Ala variant (fixed weighted mean difference −0.29 [95% CI −0.95, 0.37]; *I^2^ = *0%) (31).

### Statement 7 (Revote): Ala/Ala Carriers of *FSHR* (*FSHR* rs6165, c.919G>A, p.Thr307Ala), Which Is in Strong Linkage Disequilibrium With the Ser/Ser *FSHR* Variant (*FSHR* rs6166, c.2039A>G, p.Asn680Ser), Produce Fewer Oocytes in Response to Ovarian Stimulation Than Thr/Ala and Thr/Thr Carriers

This statement reached 52% total agreement during the first round of voting (**Figure 2**). The statement was revised to highlight that the *FSHR* variant (*FSHR* rs6165, c.919G>A) is in linkage disequilibrium (**Box 2**) with the *FSHR* rs6166 (c.2039 A>G, p.Asn680Ser), and following re-voting the revised statement reached 62% agreement (**Figure 2**); therefore, this statement did not achieve consensus. This statement received 19% disagreement from the Extended Panel, with an additional 19% of experts neither agreeing nor disagreeing with the statement. The motivations supporting these disagreements are outlined in **Supplementary Table 2**, with some experts believing there was insufficient data to support this statement and others suggesting that it depended on how the gonadotropin dose was adjusted.

Box 2 **Linkage Disequilibrium.**

- Linkage disequilibrium is defined as the non-random association of alleles of different loci that are inherited co-ordinately.
- Linkage disequilibrium differs between ethnic groups, resulting in various combinations of the different SNPs (30).
- Such genetic distinctions could potentially explain the significant disparities reported in assisted reproductive technology (ART) outcomes according to ethnicity (63) and should be taken into account when assessing studies in different populations (64).
- For example, *FSHR* rs6166 (c.2039A>G, p.Asn680Ser) and *FSHR* rs6165 (c.919G>A, p.Thr307Ala) are in linkage disequilibrium, except in some African populations (30, 31).

In a meta-analysis of five studies [including (66) and (56)] comprising 1020 women, the number of oocytes retrieved was lower in women with the Ala/Ala variant than in those with the Thr/Thr variant (fixed weighted mean difference 1.85 [95% CI 0.85, 2.85], p<0.01, Bonferroni adjusted p=0.008, *I^2^ = *0%) (31). No difference was reported between women with the Thr/Ala variant and those with the Ala/Ala variant (fixed weighted mean difference −0.37 [95% CI −1.51, 0.78]; *I^2^ = *18%) or between those with the Thr/Ala variant and those with the Thr/Thr variant (random weighted mean difference 1.62 [95% CI 0.28, 2.95], p=0.02, Bonferroni adjusted p=0.052, *I^2^ = *56%) (31). However, in contrast to this study, another study of 50 normogonadotropic women with infertility due to male factor or tubal factor in India, who were independently segregated according to genotype, reported no significant difference for the mean (SD) number of oocytes retrieved among women with different variants: Thr/Thr 16.27 (2.4), Thr/Ala 14.24 (1.2), Ala/Ala 13.86 (3.3) (66).

Two studies further categorized patients according to ovarian response based on the number of oocytes retrieved. In a prospective study of 216 Egyptian women undergoing IVF treatment for unexplained infertility, patients were classified according to ovarian response (good responders [n=111]: ≥5 oocytes retrieved; poor responders [n=105]: ≤4 oocytes retrieved). In the good ovarian responders, no statistically significant difference was observed in mean (SD) oocyte number retrieved between women with the Thr/Thr variant (13.00 [2.65]), Thr/Ala variant (11.07 [2.73]) or the Ala/Ala variant (10.20 [0.84]) (p=0.078). However, a statistically significant difference in oocyte number was reported among women with different variants in the poor ovarian responders (Ala/Ala 2.42 [0.51]; Thr/Ala 1.29 [1.14]; Thr/Thr 2.50 [0.58]; p=0.005). The Ala/Ala variant was threefold higher in poor ovarian responders compared with good ovarian responders, and the presence of a G allele significantly increased the probability of a poor ovarian response (67). Finally, in a study of 450 Chinese women who were categorized according to ovarian response (poor <5 oocytes retrieved; normal 5–14 oocytes retrieved; high >14 oocytes retrieved), the proportion with the Ala/Ala variant was significantly higher in poor ovarian responders than the proportions with the Thr/Thr variant or with the Thr/Ala variant (p<0.001) (56). 

## Statements Related to SNP of *FSHR*: rs1394205, –29G>A

### Statement 8/9 (Revote): Limited Data in Specific Ethnic Groups Suggest That A/A Allele Carriers of the *FSHR* −29 Variant (rs1394205, −29G>A) Require Higher Amounts of Gonadotropin and Produce Fewer Oocytes in Response to Ovarian Stimulation than G/G Carriers

Statements 8 and 9 each received 50% total agreement in the first round of voting (**Figure 2**). The same concerns were identified for each statement and so they were softened and merged with the specification that the effect can be seen in ‘at least some ethnic groups’ for the second round of voting, during which 76% total agreement was reached. The reasons given by participants for disagreeing with this statement are shown in **Supplementary Table 2**. One participant explained that ethnicity had been shown to play a fundamental role in SNPs and that 1% of the population would need to carry the *FSHR* −29 variant in order for it to have a clinical application, and another participant stated that the studies supporting this statement had not been correctly planned.

The *FSHR* −29 variant (rs1394205, −29G>A) has been associated with reduced transcriptional activity, primary or secondary amenorrhea, and poor response to exogenous FSH (31, 59, 68). The latter has not been found consistently in other studies (59). There is controversial evidence for the influence of this variant on ovarian stimulation. Studies suggest that carriers of the A allele, which has a frequency of 49–55% in a Hispano-American population (59), have reduced *FSHR* expression, are more likely to have a poor ovarian response (31, 68) and may require a higher amount of FSH during OS; exogenous FSH consumption was significantly higher (p<0.001) in A/A carriers than in G/G or G/A carriers in a retrospective study of 50 women undergoing ART (69). A meta-analysis of three studies (n=709 women) also found that A/A carriers needed significantly higher doses of FSH during OS (31). These studies were conducted in only a few homogenous populations where the polymorphism has been found to be highly prevalent (31) and the results may not be generalizable to different populations.

This variant may be associated with fewer oocytes being retrieved during ART. A review of three studies reporting on this polymorphism showed that a lower number of oocytes were retrieved in A/A carriers than A/G or G/G carriers, although the association was not found to be significant (31). One of these studies reported that the mean (SD) number of oocytes retrieved was significantly lower in A/A carriers (6.00 [1.09]) than in G/G carriers (17.88 [1.75]; P = 0.003) (66). A study by Desai et al. (70) found that the mean (SD) number of oocytes retrieved in A/A carriers (10.50 [1.19]) was significantly lower when compared to G/G carriers (16.43 [1.50]; p=0.046) (70). This association has also been found in another study by the same group (68), who reported that increasing the dose of exogenous FSH did not improve oocyte development, probably due to insufficiency of *FSHR* expression in granulosa cells (68). These studies from one group were restricted to a small, homogenous population, and further studies in mixed populations are needed to generalize the results.

## Statements Related to SNP of *FSHB*: rs10835638, −211G>T

### Statement 10: The Data on an Association of *FSHB* (*FSHB* rs10835638, −211G>T) With Basal Levels of FSH and Production of Oocytes in Response to Ovarian Stimulation Are Contradictory

This statement reached 90% agreement during the first round of voting (**Figure 2**). The consensus participants did not give any reasons for disagreeing with this statement (**Supplementary Table 2**).

This statement was supported by evidence from three studies (45–47). A cross-sectional study evaluated the potential effects of the *FSHB* rs10835638 (−211G>T) variant on hormonal profiles and IVF/ICSI outcomes in 140 normo-ovulatory women in Brazil (47). The distributions of the GG (wild-type) variant (n=102) and GT variant (n=38) were 86.4% and 13.6%, respectively; the TT variant was not detected in any women. A poor response to ovarian stimulation was more common in women with the GT variant compared with those with the GG variant (47.4% *vs*. 26.5%; p=0.010), and fewer oocytes were retrieved from those with the GT variant than from those with the GG variant (3.0 *vs*. 5.0; p=0.03). However, no difference in pregnancy rates were reported between women with different variants.

Two studies assessing the effects of *FSHB* rs10835638 on basal FSH levels, in women with known infertility compared with a control group of healthy women, reported that women expressing the T allele showed significantly higher FSH basal levels. One study reported that the T allele was associated with significantly higher basal FSH levels in both non-pregnant healthy women (n=169) and female partners in infertile couples (n=186) (T-allele effect: 0.80 IU/L, p=0.001 after Bonferroni testing) (46). *FSHB* rs10835638 was estimated to explain 3.5% of the total variance of the measured serum FSH levels in healthy women and 1.6% in the female partners of infertile couples, and could have a diagnostic value in fertility clinics to detect female patients with genetically inherited elevated basal FSH and LH levels (46). In the other study, eumenorrheic women attending an IVF unit for predominantly male-factor infertility (n=365) were compared with a control group of women with proven fertility (n=438) (45). The distribution of the variants was 2.5% for the TT variant (n=9), 23.8% for the GT variant (n=87) and 73.7% (n=269) for the GG variant. The TT variant was strongly associated with an elevated mean (SD) basal FSH (TT 9.6 [2.4] U/L *vs*. GT 7.4 [1.8] U/L *vs*. GG 7.7 [2.2] U/L; TT-homozygosity effect 2.05 U/L, p=0.003) (45).

## Statements Related to SNPs of the *LHB/LHCGR* Genes

### Statement 11: Limited Data Suggest That Polymorphisms of the *LHB/LHCGR* Genes (*V-betaLH* rs1800447, c.82T>C, p.Trp8Arg; *V-betaLH* rs34349826, c.104 A>G, p.Ile35Thr; *LHCGR* rs2293275, c.935A>G, p.Asn312Ser) Can Influence Ovarian Stimulation Outcomes and May Represent Targets for Pharmacogenomic Research in ART

This statement reached 80% agreement during the first round of voting (**Figure 2**). The reason given by participants for disagreeing with this statement was that it had not been proven (**Supplementary Table 2**).

Four studies supported this statement, the collective results of which suggest that clinicians should be aware of patients with *LHB* polymorphisms, who may, consequently, fail to respond to ovarian stimulation (43, 71–73).

Hypo-sensitivity to exogenous FSH was observed in a retrospective study of 220 normogonadotropic Danish women undergoing controlled ovarian stimulation who were carriers of *V-betaLH* (rs1800447 [c.82 T>C, p.Trp8Arg] and rs34349826 [c.104 A>G, p.Ile135Thr] polymorphisms), which are common genetic variants of *LHB* (43). Daily doses of r-hFSH were administered on an individualized basis, tailored to age, body mass index, baseline FSH, and antral follicle count. The *LHB* genotype was assessed by immunofluorometric assay. A total of 24 women carried the *V-betaLH* variant, of whom 21 were heterozygous and three were homozygous; 196 of the women were wild type. The differences in the mean number of oocytes retrieved and the fertilization and pregnancy rates for each cycle were not statistically significant between the *V-betaLH* genotypes, but carriers of *V-betaLH* variant received a significantly higher cumulative dose of r-hFSH compared with women with wild type LH (2435.86 ± 932.8 IU *vs*. 1959.8 ± 736.45 IU; p=0.048). A within-design one-way ANOVA analysis showed that the *V-betaLH* variants had a statistically significant effect (p<0.01) on the cumulative dose of r-hFSH, with a mean (SD) increase from 1959.8 (736.45) IU for wild type carriers, to 2267.5 (824.3) IU and 3558.3 (970.9) IU, for heterozygotic and homozygotic carriers, respectively. These results confirm that carriers of *V-betaLH* variants have hypo-sensitivity to exogenous FSH during controlled ovarian stimulation (43).

In their systematic review of the current status of pharmacogenetic analyses of controlled ovarian hyperstimulation, Altmäe et al. concluded that there is accumulating data to suggest that the ovarian response to COH is mediated by various polymorphisms, including variants of the *V-betaLH* and *LHCGR* genes (72). However, further studies investigating the predictive value of such genetic polymorphisms as markers of COH in subgroups of women who may require supplementation with exogenous LH during ovarian stimulation are needed (72). In addition, the results from an observational preliminary trial of 60 normogonadotropic patients undergoing IVF/ICSI suggested that women with the most common polymorphism of *LHB* (*V-betaLH*) were hyporesponsive to r-hFSH (72). A greater proportion (31.8%) of carriers of *V-betaLH* were identified among 22 women who required a cumulative dose of r-hFSH of >3500 IU, relative to those who required between 2000 and 3500 IU r-hFSH (one woman [6.7%] *V-betaLH* of 15 women). *V-betaLH* variants were detected in 23 women who required <2000 IU r-hFSH (73). Lastly, sequence analysis indicated that heterozygous point mutations in the *LHB* gene (Trp8Arg and Ile15Thr) were present in a 35-year-old woman who had failed to conceive after six cycles of human menopausal gonadotrophin (hMG) therapy for ovarian induction (71). The observed LH hypersecretion was likened to that seen in PCOS. A further cycle of ovarian stimulation with hMG, during which estrogen–progesterone replacement therapy effectively controlled basal LH and FSH, led to successful conception and delivery outcomes (71).

## Discussion

This Delphi consensus provides clinical perspectives from a diverse international group of experts. It generates a series of literature-supported consensus statements regarding the influence of specific gonadotropin and gonadotropin receptor variants on clinical ovarian stimulation outcomes that will be useful to optimize current stimulation protocols. The consensus results suggest that there is evidence to support a link between SNP variants in gonadotropin and gonadotropin receptors and ovarian stimulation outcomes, although data for some variants are still lacking.

Our consensus demonstrates that polymorphisms of gonadotropins (FSH and LH) and their receptors may impact ovarian stimulation in a number of ways. SNP of *FSHR* have been shown to influence basal FSH levels (*FSHR* rs6166, c.2039A>G, p.Asn680Ser), gonadotropin consumption (*FSHR* rs6166, c.2039A>G, p.Asn680Ser; *FSHR* rs1394205, −29G>A), oocyte number (*FSHR* rs6166, c.2039A>G, p.Asn680Ser; *FSHR* rs1394205, −29G>A), and may affect duration of gonadotropin stimulation (*FSHR* rs6165, c.919G>A, p.Thr307Ala) and risk of OHSS (*FSHR* rs6166, c.2039A>G, p.Asn680Ser). Evidence supporting an association between SNP of *FSHB* (rs10835638, −211G>T) and basal FSH levels or oocyte number are currently limited. Furthermore, our consensus highlights that there are limited data that polymorphisms of the *LHB/LHCGR* genes can influence ovarian stimulation outcomes and could potentially represent future targets for pharmacogenomic research in ART.

Although there is strong supporting evidence for the impact of polymorphisms of *FSHR* on several outcomes, this Delphi Consensus shows there are only modest data on the clinical relevance that would support these polymorphisms as the basis for pharmacogenetic approaches to treatment, although we acknowledge that this is an area where new data are being published. A recent multicentre, multinational, prospective study in 368 predicted normal responder women from Vietnam, Belgium, and Spain, published since the selection of the literature to be considered in this Delphi consensus, reported only minimal clinical impact of genotyping for *FSHR* SNPs rs6165 (c.919G>A, p.Thr307Ala), rs6166 (c.2039A>G, p.Asn680Ser), rs1394205 (−29G>A) and *FSHB* SNP rs10835638 (−211G>T) prior to initiating ovarian stimulation with r-hFSH (74). Although the study reported a significantly lower number of oocytes retrieved in heterozygous patients for the *FSHR* variants rs6166 and rs1394205, as well as a significantly higher rate of hypo-response in heterozygous patients for the *FSHR* variant rs6166, this resulted in a change of just one or two oocytes in a population of normal responders (74).

Currently, there is contradictory evidence for the clinical impact of polymorphisms in *FSHB* on ovarian stimulation. Three studies supporting this consensus reported that women expressing the T-allele of the *FSHB* rs10835638 (−211G>T) polymorphism had fewer retrieved oocytes (47) and significantly higher basal FSH levels compared with women carrying the wild type (GG) variant (45, 46). However, in contrast to the aforementioned studies, La Marca et al. observed that *FSHB* rs10835638 does not affect FSH basal levels *per se*. The authors observed a significant reduction of FSH basal levels in women expressing the T-allele compared with the wild-type genotype (75).

The inconsistency between some studies, which is also highlighted in the Alviggi 2018 systematic review and meta-analysis (31), is likely due to differences in inclusion criteria between studies, as well as the use of different gonadotropin products and doses, and allowance for dose adjustments during treatment, highlighting the need for new prospective studies in this field.

As already stated in this Delphi consensus, there are limited data on the influence of polymorphisms of the *LHB/LHCGR* genes on ovarian stimulation outcomes and their usefulness in pharmacogenomic research in ART. While the usefulness of polymorphisms in these genes *per se* remains to be determined, a recent study (also published since the selection of the literature for consideration in this Delphi Consensus) may indicate some clinical value in determining the need for LH supplementation compared with current supplementation protocols, reporting higher clinical pregnancy rates (p=0.049) and a trend towards improved live birth rates (p=0.082) in 193 women when supplementation was based on a woman’s SNP profile compared with conventional methods (76). Furthermore, in a cross-sectional study of *LHCGR* rs2293275 (c.935 A>G, p.Asn312Ser), Lindgren et al. reported no significant difference in the number of oocytes retrieved or obvious differences in embryo quality between Ser/Ser, Asn/Ser or Asn/Asn carriers. However, significantly higher clinical pregnancy rates were reported in Ser/Ser carriers compared with Asn/Asn carriers (OR 1.61 [95% CI 1.13, 2.29], p=0.008) (41). These studies suggest that individualising protocols based on specific genotypes, rather than the number and morphological characteristics of the embryos retrieved, may be beneficial in terms of improved pregnancy rates.

### Strengths

This consensus has a number of strengths, including the fact that each of the statements in the consensus were supported by a number of peer-reviewed studies. Furthermore, the majority of women included in these studies were aged <35 years (mean/median age ~32 years). As advanced maternal age (>35 years) is associated with a reduction in ovarian reserve, oocyte/embryo competence and cumulative live birth rate (77–79), the fact that most women in these studies were aged <35 years suggests that age was less likely to impact ovarian response, making it easier to discern the influence of SNP variants. Another strength was that the participants of the consensus were fertility experts from across the globe, representing different regions, including Europe, Asia, and South America, reflecting the quality of healthcare and different approaches to infertility treatment in different parts of the world.

### Limitations

The consensus does have some limitations that should be acknowledged. Firstly, the consensus does not represent an exhaustive list of statements referring to all polymorphisms potentially affecting ovarian stimulation. Furthermore, the statements and supporting literature were chosen according to the expert opinion of the Scientific Coordinators and the Scientific Board, and the inclusion of the statements and supporting literature was based on the expert opinion of the Extended Panel. Another limitation was that none of the statements reached 100% agreement, with most statements reaching consensus even though some participants disagreed with them, and only three statements (Statements 1, 10 and 11) achieved a high level of agreement (defined as ≥80% of participants voting ‘agree’ or ‘absolutely agree’). Furthermore, not all statements reached consensus, with Statements 4 and 7 failing to reach consensus even after the statements were revised and revoted. The main reasons stated by participants for disagreeing with a given statement included a limited number of studies, small sample sizes, poor replication of data between studies, and the fact that most data were from observational studies based on homogeneous populations and with homogeneous protocols of ovarian stimulation. Furthermore, a number of participants cited the absence of data from RCTs as a reason for disagreeing with a given statement.

### Future Research

Our consensus represents an opportunity to encourage researchers to initiate studies to verify whether a pharmacogenomic approach (i.e. the individualization of treatment based on a patient’s genetic profile) could lead to an improved patient-tailored approach to ovarian stimulation. This could be beneficial, as a more unified approach among studies will enable us to reach future clinical decisions. Given the lack of studies on pharmacogenomic approaches, the consensus was unable to include any statement(s) concerning a pharmacogenomic approach to ovarian stimulation. Conversely, we believe that there is sufficient evidence from the results of our consensus to support the fact that specific genetic variants could represent interesting causative factors of impaired ovarian response that cannot be explained by other parameters such as ovarian reserve markers. Gene association studies could focus on all SNPs known to influence ovarian response (including those highlighted in this Delphi consensus), in order to examine whether an individualized treatment approach may significantly improve ovarian stimulation outcomes, compared with a standard approach. Ideally, the ovarian response would be correlated with the “global genetic background” assessed by SNP genotyping, exome sequencing or even whole genome sequencing combined with artificial intelligence in large, multicenter studies. Greater efforts should focus on increasing the number of observations, for example, by utilizing large-scale datasets such as the UKBioBank (80). Hypothetically, the development of a large international registry concerning genome-wide association studies in IVF could also be beneficial. This strategy would lead to “real-world” data that could provide interesting findings concerning the importance of genetics in Reproductive Genetics and ART (81, 82). Nonetheless, the quality of association studies in Reproductive Genetics should take into account selection bias, different treatments and differential follow-ups among IVF centers. In addition, the use of check-list proposed STROBE or GRACE guidelines should be encouraged to increase the overall quality of published articles (21, 83, 84).

Specifically, further research could include investigating an association between *FSHR* rs6166 (c.2039A>G, p.Asn680Ser) and ovarian morphology, AFC following ovarian stimulation and basal AFC in different age groups. A multivariate analysis assessing the impact of r-hLH supplementation according to SNP status (specifically, *FSHR* rs6166 [c.2039A>G, p.Asn680Ser] and *LHCGR* rs2293275 [c.935A>G, p.Asn312Ser]) would also be relevant, as would investigating the effect of follicular fluid steroid hormone levels in different *FSHR* and *LHCGR* SNPs and their implication as a biomarker for oocyte preservation in women postponing pregnancy. Furthermore, it would be of interest to test the hypothesis that different r-hFSH doses with or without r-hLH supplementation may improve ongoing pregnancy and live birth rates per started cycle compared with a standard dose, taking into account age and ovarian reserve, and according to SNP status.

## Conclusions

This Delphi consensus supports a link between some genetic variants in gonadotropin and gonadotropin receptors and ovarian stimulation outcomes. The consensus results reinforce the idea that pharmacogenomics may provide a promising new field examining genotype-specific responses to ovarian stimulation medication that may help tailor ovarian stimulation therapies to individual patients, optimizing ART success outcomes.

## Data Availability Statement

The original contributions presented in the study are included in the article/**Supplementary Material**. Further inquiries can be directed to the corresponding author.

## Ethics Statement

Written informed consent was obtained from all Consensus participants for the publication of their name in **Table 2**.

## Author Contributions

AC and FT were Scientific Coordinators for the Delphi consensus; AC, FT CA, HMB, RF, LH, NPP, GARR, MS, and SKS were members of the Scientific Board. One Scientific Board member declined authorship of the article owing to personal reasons. All authors made substantial contributions to the concept and design, or the acquisition, analysis or interpretation of data, and to the drafting of the manuscript or revising it critically for important intellectual content. In addition, all authors provided final approval of the manuscript.

## Funding

The work was funded by Merck KGaA, Darmstadt, Germany.

## Conflict of Interest

The Delphi consensus was coordinated by a healthcare consulting and training company (Sanitanova Srl, Milan, Italy). The consensus concept was initiated and funded by Merck KGaA, Darmstadt, Germany. The sponsor was involved early in the process, defining the overarching topic to be discussed, but did not participate in the development of the statements or in any of the meetings or discussions involved in developing the Delphi consensus. The statements were, therefore, developed independently of the industry sponsor. The authors from Merck KGaA, Darmstadt, Germany, were only involved in the development of the manuscript, critically revising it for important intellectual content, especially in the Introduction, Results and Discussion sections, but could not alter the consensus statements in any way.

DC, TD’H, and SL are employees of Merck KGaA, Darmstadt, Germany. AC has received of honoraria and consultation from Merck KGaA, Darmstadt, Germany and Event Planet SpA. CA has received of honoraria for lectures from Merck KGaA, Darmstadt, Germany and Event Planet SpA. HB has been scientific advisor for Merck KGaA, Darmstadt, Germany and MSD. RF has received honoraria from Merck KGaA, Darmstadt, Germany and affiliates for lectures. NP received research grants or honoraria for lectures from: Merck KGaA, Darmstadt, Germany, MSD, Ferring Pharmaceuticals, Besins International, Roche Diagnostics, IBSA, Theramex, Gedeon Richter. MS received honoraria and research grants from Merck KGaA, Darmstadt, Germany, Ferring and IBSA. SS was a speaker at non-promotional educational symposia by Merck KGaA, Darmstadt, Germany and Ferring, and received independent research grants from Merck KGaA, Darmstadt, Germany and Ferring.

The remaining author declares that the research was conducted in the absence of any commercial or financial relationships that could be construed as a potential conflict of interest.

## Publisher’s Note

All claims expressed in this article are solely those of the authors and do not necessarily represent those of their affiliated organizations, or those of the publisher, the editors and the reviewers. Any product that may be evaluated in this article, or claim that may be made by its manufacturer, is not guaranteed or endorsed by the publisher.
