# Supplementary material for: Effect of Genetic Variants of Gonadotropins and Their Receptors on Ovarian Stimulation Outcomes: A Delphi Consensus
Source: Front Endocrinol (Lausanne). 2022 Feb 1;12:797365. doi: 10.3389/fendo.2021.797365 (PMC8844496; doi:10.3389/fendo.2021.797365)
Supplement: Supplementary file 1 [file Table_1.docx]

Supplementary Material

## *Supplementary Table 1*

| **Name** | **Publications in Reproductive Genetics (2018 to 2020)** |
| --- | --- |
| **Scientific Coordinators** | |
| Alessandro Conforti | Cariati F, Carbone L, Conforti A, Bagnulo F, Peluso SR, Carotenuto C, Buonfantino C, Alviggi E, Alviggi C, Strina I (2020). Bisphenol A-induced epigenetic changes and its effects on the male reproductive system. Front Endocrinol. **11**:453. doi:10.3389/fendo.2020.00453.  Casella C, Carbone L, Conforti A, Marrone V, Cioffi G, Buonfantino C, De Rosa P, Avino L, Capalbo A, Alviggi C, Di Lorenzo P (2020). Preimplantation genetic testing: Comparative analysis of jurisprudential regulations. Ital J Gynaecol Obstet. **32**(4):237–47. doi:10.36129/jog.32.04.03.  Conforti A, Vaiarelli A, Cimadomo D, Bagnulo F, Peluso S, Carbone L, Rella FD, De Placido G, Ubaldi FM, Huhtaniemi I, Alviggi C (2019). Pharmacogenetics of FSH action in the female. Front Endocrinol. **10**:398. doi:10.3389/fendo.2019.00398.  Alviggi C, Conforti A, Santi D, Esteves SC, Andersen CY, Humaidan P, Chiodini P, De Placido G, Simoni M (2018). Clinical relevance of genetic variants of gonadotrophins and their receptors in controlled ovarian stimulation: a systematic review and meta-analysis. Hum Reprod Update. **24**(5):599–614. doi:10.1093/humupd/dmy019. |
| Frank Tüttelmann | Sansone A, Schubert M, Tüttelmann F, Krallmann C, Zitzmann M, Kliesch S, Gromoll J (2021). Pituitary response to GnRH stimulation tests in different FSHB-211 G/T genotypes. Hum Reprod. **36**(5):1376–82. doi:10.1093/humrep/deab033.*  Araujo TF, Friedrich C, Grangeiro CHP, Martelli LR, Grzesiuk JD, Emich J, Wyrwoll MJ, Kliesch S, Simões AL, Tüttelmann F (2020). Sequence analysis of 37 candidate genes for male infertility: challenges in variant assessment and validating genes. Andrology. **8**(2):434–41. doi:10.1111/andr.12704.  Krausz C, Riera-Escamilla A, Moreno-Mendoza D, Holleman K, Cioppi F, Algaba F, Pybus M, Friedrich C, Wyrwoll MJ, Casamonti E, Pietroforte S, Nagirnaja L, Lopes AM, Kliesch S, Pilatz A, Carrell DT, Conrad DF, Ars E, Ruiz-Castañé E, Aston KI, Baarends WM, Tüttelmann F (2020). Genetic dissection of spermatogenic arrest through exome analysis: clinical implications for the management of azoospermic men. Genet Med. **22**(12):1956–66. doi:10.1038/s41436-020-0907-1.  Krenz H, Gromoll J, Darde T, Chalmel F, Dugas M, Tüttelmann F (2020). The Male Fertility Gene Atlas: a web tool for collecting and integrating OMICS data in the context of male infertility. Hum Reprod. **35**(9):1983–90. doi:10.1093/humrep/deaa155.  Rudnik-Schöneborn S, Tüttelmann F, Zschocke J (2020). Genetic diagnosis prior to assisted reproduction: recommendations of the new 2019 S2k guideline. Gynakologische Endokrinologie. **18**(2):97–107. doi:10.1007/s10304-020-00317-y.  Schilit SLP, Menon S, Friedrich C, Kammin T, Wilch E, Hanscom C, Jiang S, Kliesch S, Talkowski ME, Tüttelmann F, MacQueen AJ, Morton CC (2020). SYCP2 translocation-mediated dysregulation and frameshift variants cause human male infertility. Am J Hum Genet. **106**(1):41–57. doi:10.1016/j.ajhg.2019.11.013.  Schuppe H-C, Pilatz A, Fietz D, Diemer T, Köhn FM, Tüttelmann F, Kliesch S (2020). Infertility due to azoospermia: Differential diagnosis, genetic aspects, histopathology of the testis, and surgical sperm retrieval. J Urol Urogynakologie. **27**(3):102–12. doi:10.1007/s41972-020-00111-9.  Wistuba J, Beumer C, Warmeling AS, Sandhowe-Klaverkamp R, Stypmann J, Kuhlmann M, Holtmeier R, Damm OS, Tüttelmann F, Gromoll J (2020). Testicular blood supply is altered in the 41,XX^Y^* Klinefelter syndrome mouse model. Sci Rep. **10**(1):14369. doi: 10.1038/s41598-020-71377-0.  Wyrwoll MJ, Temel ŞG, Nagirnaja L, Oud MS, Lopes AM, van der Heijden GW, Heald JS, Rotte N, Wistuba J, Wöste M, Ledig S, Krenz H, Smits RM, Carvalho F, Gonçalves J, Fietz D, Türkgenç B, Ergören MC, Çetinkaya M, Başar M, Kahraman S, McEleny K, Xavier MJ, Turner H, Pilatz A, Röpke A, Dugas M, Kliesch S, Neuhaus N; GEMINI Consortium, Aston KI, Conrad DF, Veltman JA, Friedrich C, Tüttelmann F (2020). Bi-allelic mutations in M1AP are a frequent cause of meiotic arrest and severely impaired spermatogenesis leading to male infertility. Am J Hum Genet. **107**(2):342-351. doi:10.1016/j.ajhg.2020.06.010.  Busch AS, Tüttelmann F, Cremers JF, Schubert M, Nordhoff V, Schüring AN, Zitzmann M, Gromoll J, Kliesch S (2019). FSHB −211 G>T Polymorphism as predictor for TESE success in patients With unexplained azoospermia. J Clin Endocrinol Metab. **104**(6):2315–24. doi:10.1210/jc.2018-02249.  Darde TA, Lecluze E, Lardenois A, Stévant I, Alary N, Tüttelmann F, Collin O, Nef S, Jégou B, Rolland AD, Chalmel F (2019). The ReproGenomics Viewer: a multi-omics and cross-species resource compatible with single-cell studies for the reproductive science community. Bioinformatics. **35**(17):3133–9. doi:10.1093/bioinformatics/btz047.  Toth B, Baston-Büst DM, Behre HM, Bielfeld A, Bohlmann M, Bühling K, Dittrich R, Goeckenjan M, Hancke K, Kliesch S, Köhn FM, Krüssel J, Kuon R, Liebenthron J, Nawroth F, Nordhoff V, Pinggera GM, Rogenhofer N, Rudnik-Schöneborn S, Schuppe HC, Schüring A, Seifert-Klauss V, Strowitzki T, Tüttelmann F, Vomstein K, Wildt L, Wischmann T, Wunder D, Zschocke J (2019). Diagnosis and treatment before assisted reproductive treatments. Guideline of the DGGG, OEGGG and SGGG (S2k Level, AWMF Register Number 015-085, February) - Part 2, Hemostaseology, Andrology, Genetics and History of Malignant Disease. Geburtshilfe Frauenheilkd. **79**(12):1293–1308. doi:10.1055/a-1017-3478.  Tüttelmann F, Ruckert C, Röpke A (2018). Disorders of spermatogenesis: Perspectives for novel genetic diagnostics after 20 years of unchanged routine. Med Genet. **30**(1):12–20. doi:10.1007/s11825-018-0181-7.  Busch AS, Kliesch S, Tüttelmann F, Gromoll J (2015). FSHB −211G>T stratification for follicle-stimulating hormone treatment of male infertility patients: making the case for a pharmacogenetic approach in genetic functional secondary hypogonadism. Andrology. **3**(6):1050–3. doi: 10.1111/andr.12094.*  Schüring AN, Busch AS, Bogdanova N, Gromoll J, Tüttelmann F (2013). Effects of the FSH-β-subunit promoter polymorphism –211G->T on the hypothalamic-pituitary-ovarian axis in normally cycling women indicate a gender-specific regulation of gonadotropin secretion. J Clin Endocrinol Metab. **98**(1):E82–6. doi:10.1210/jc.2012-2780.*  Tüttelmann F, Laan M, Grigorova M, Punab M, Sõber S, Gromoll J (2012). Combined effects of the variants FSHB –211G>T and FSHR 2039A>G on male reproductive parameters. J Clin Endocrinol Metab. **97**(10):3639–47. doi:10.1210/jc.2012-1761.* |
| **Scientific Board** | |
| Carlo Alviggi | Cariati F, Carbone L, Conforti A, Bagnulo F, Peluso SR, Carotenuto C, Buonfantino C, Alviggi E, Alviggi C, Strina I (2020). Bisphenol A-induced epigenetic changes and its effects on the male reproductive system. Front Endocrinol (Lausanne). **11**:453. doi: 10.3389/fendo.2020.00453.  Casella C, Carbone L, Conforti A, Marrone V, Cioffi G, Buonfantino C, De Rosa P, Avino L, Capalbo A, Alviggi C, Di Lorenzo P (2020). Preimplantation genetic testing: Comparative analysis of jurisprudential regulations. Ital J Gynaecol Obstet. **32**(4): 237–47. doi:10.36129/jog.32.04.03.  Conforti A, Vaiarelli A, Cimadomo D, Bagnulo F, Peluso S, Carbone L, Di Rella F, De Placido G, Ubaldi FM, Huhtaniemi I, Alviggi C (2019). Pharmacogenetics of FSH Action in the Female. Front Endocrinol (Lausanne). **10**:398. doi:10.3389/fendo.2019.00398.  Alviggi C, Conforti A, Santi D, Esteves SC, Andersen CY, Humaidan P, Chiodini P, De Placido G, Simoni M (2018). Clinical relevance of genetic variants of gonadotrophins and their receptors in controlled ovarian stimulation: a systematic review and meta-analysis. Hum Reprod Update. **24**(5):599–614. doi:10.1093/humupd/dmy019. |
| Hermann M. Behre | Greither T, Schumacher J, Dejung M, Behre HM, Zischler H, Butter F, Herlyn H (2020). Fertility relevance probability analysis shortlists genetic markers for male fertility impairment. Cytogenet Genome Res. **160**(9):506–22. doi:10.1159/000511117.  Toth B, Baston-Büst DM, Behre HM, Bielfeld A, Bohlmann M, Bühling K, Dittrich R, Goeckenjan M, Hancke K, Kliesch S, Köhn FM, Krüssel J, Kuon R, Liebenthron J, Nawroth F, Nordhoff V, Pinggera GM, Rogenhofer N, Rudnik-Schöneborn S, Schuppe HC, Schüring A, Seifert-Klauss V, Strowitzki T, Tüttelmann F, Vomstein K, Wildt L, Wischmann T, Wunder D, Zschocke J (2019). Diagnosis and treatment before assisted reproductive treatments. guideline of the DGGG, OEGGG and SGGG (S2k Level, AWMF Register Number 015-085, February 2019) - Part 2, Hemostaseology, Andrology, Genetics and History of Malignant Disease. Geburtshilfe Frauenheilkd. **79**(12):1293–1308. doi:10.1055/a-1017-3478.  Giebler M, Greither T, Behre HM (2018). Differential regulation of *PIWI-LIKE 2* expression in primordial germ cell tumor cell lines by promoter methylation. Front Genet. **9**:375. doi: 10.3389/fgene.2018.00375.  Giebler M, Greither T, Müller L, Mösinger C, Behre HM (2018). Altered *PIWI-LIKE 1* and *PIWI-LIKE 2* mRNA expression in ejaculated spermatozoa of men with impaired sperm characteristics. Asian J Androl. **20**(3):260–4. doi:10.4103/aja.aja_58_17. |
| Robert Fischer | Fischer R, Nakano FY, Roque M, Bento FC, Baukloh V, Esteves SC (2019). A quality management approach to controlled ovarian stimulation in assisted reproductive technology: the "Fischer protocol". Panminerva Med. **61**(1):11–23. doi: 10.23736/S0031-0808.18.03549-8. |
| José Gonçalves Franco Junior | Franco JG, Dieamant F, Oliveira JBA (2020). Noninvasive preimplantation genetic testing for aneuploidies (NiPGT-A) and the principle of primum non nocere. J Bras Reprod Assist. **24**(4):391–3. doi:10.5935/1518-0557.20200075.  Franco JG, Vagnini LD, Petersen CG, Renzi A, Canas MCT, Petersen B, Ricci J, Nicoletti A, Zamara C, Dieamant F, Oliveira JBA (2020). Noninvasive preimplantation genetic testing for aneuploidy (niPGT-A): The first Brazilian baby. J Bras Reprod Assist. **24**(4):1. doi:10.5935/1518-0557.20200074.  Vagnini LD, Petersen CG, Renzi A, Dieamant F, Oliveira JBA, Oliani AH, Canas MCT, Nakano R, Almodin CG, Marcondes C, Ceschin A, Amaral A, Soares JB, Lopes J, Franco AC, Franco JG (2020). Relationship between age and blastocyst chromosomal ploidy analyzed by noninvasive preimplantation genetic testing for aneuploidies (NiPGT-A). J Bras Reprod Assist. **24**(4): 395–9. doi:10.5935/1518-0557.20200061.  Franco, J.G (2019). New perspectives with the use of noninvasive chromosome screening (NICS) in ART. J Bras Reprod Assist. **23**(4):321–2. doi:10.5935/1518-0557.20190071.  Oliveira JBA, Petersen CG, Mauri AL, Vagnini LD, Renzi A, Petersen B, Mattila M, Dieamant F, Baruffi RLR, Franco JG (2018). Association between body mass index and sperm quality and sperm DNA integrity. A large population study. Andrologia. **50**(3):e12889. doi:10.1111/and.12889.  Petersen CG, Mauri AL, Vagnini LD, Renzi A, Petersen B, Mattila M, Comar V, Ricci J, Dieamant F, Oliveira JBA, Baruffi RLR, Franco JG (2018). The effects of male age on sperm DNA damage: An evaluation 2,178 semen samples. J Bras Reprod Assist. **22**(4):323–30. doi:10.5935/1518-0557.20180047. |
| Liang Hu | Zheng W, Zhou Z, Sha Q, Niu X, Sun X, Shi J, Zhao L, Zhang S, Dai J, Cai S, Meng F, Hu L, Gong F, Li X, Fu J, Shi R, Lu G, Chen B, Fan H, Wang L, Lin G, Sang Q (2020). Homozygous mutations in BTG4 cause zygotic cleavage failure and female infertility. Am J Hum Genet. **107**(1):24–33. doi:10.1016/j.ajhg.2020.05.010.  Dai C, Chen Y, Hu L, Du J, Gong F, Dai J, Zhang S, Wang M, Chen J, Guo J, Zheng W, Lu C, Wu Y, Lu G, Lin G (2019). ZP1 mutations are associated with empty follicle syndrome: evidence for the existence of an intact oocyte and a zona pellucida in follicles up to the early antral stage. A case report. Hum Reprod. **34**(11):2201–7. doi: 10.1093/humrep/dez174.  Liu L, Leng L, Liu C, Lu C, Yuan Y, Wu L, Gong F, Zhang S, Wei X, Wang M, Zhao L, Hu L, Wang J, Yang H, Zhu S, Chen F, Lu G, Shang Z, Lin G (2019). An integrated chromatin accessibility and transcriptome landscape of human pre-implantation embryos. Nat Commun. **10**(1):364. doi:10.1038/s41467-018-08244-0.  Luo K, Lan Y, Xie P, Gong F, Xiong B, Tan Y, Zhou S, Yang Z, Lin G, Hu L (2019). Next-generation sequencing analysis of embryos from mosaic patients undergoing in vitro fertilization and preimplantation genetic testing. Fertil Steril. **112**(2):291–7.e3. doi:10.1016/j.fertnstert.2019.03.035.  Xie P, Hu L, Tan Y, Gong F, Zhang S, Xiong B, Peng Y, Lu GX, Lin G (2019). Retrospective analysis of meiotic segregation pattern and interchromosomal effects in blastocysts from inversion preimplantation genetic testing cycles. Fertil Steril. **112**(2):336–42.e3. doi:10.1016/j.fertnstert.2019.03.041.  Zhou S, Cheng D, Ouyang Q, Xie P, Lu C, Gong F, Hu L, Tan Y, Lu G, Lin G (2018). Prevalence and authenticity of de-novo segmental aneuploidy (>16 Mb) in human blastocysts as detected by next-generation sequencing. Reprod Biomed Online. **37**(5):511–20. doi:10.1016/j.rbmo.2018.08.006. |
| Nikolaos P. Polyzos | Neves AR, Devesa M, Martínez F, Garcia-Martinez S, Rodriguez I, Polyzos NP, Coroleu B (2020). What is the clinical impact of the endometrial receptivity array in PGT-A and oocyte donation cycles? Obstet Gynecol Surv. **75**(1):36–7. doi:10.1097/01.ogx.0000652496.75061.7a.  Neves AR, Devesa M, Martínez F, Garcia-Martinez S, Rodriguez I, Polyzos NP, Coroleu B (2019). What is the clinical impact of the endometrial receptivity array in PGT-A and oocyte donation cycles? J Assist Reprod Genet. **36**(9):1901–8. doi:10.1007/s10815-019-01535-5. |
| Achyuta Gottumukkala Rama Raju | Ramaraju GA, Cheemakurthi R, Kalagara M, Prathigudupu K, Balabomma KL, Mahapatro P, Thota S, Kommaraju AL, Muvvala SPR (2021). Effect of LHCGR Gene Polymorphism (rs2293275) on LH Supplementation Protocol Outcomes in Second IVF Cycles: A Retrospective Study. Front Endocrinol (Lausanne). 12: 628169.  Ramaraju GA, Cheemakurthi R, Prathigudupu K, Balabomma KL, Kalagara M, Thota S, Kota M (2018). Role of LH polymorphisms and r-hLH supplementation in GnRH agonist treated ART cycles: A cross sectional study. Eur J Obstet Gynecol Reprod Biol. **222**:119–25. doi:10.1016/j.ejogrb.2018.01.025. |
| Manuela Simoni | Alviggi C, Conforti A, Santi D, Esteves SC, Andersen CY, Humaidan P, Chiodini P, De Placido G, Simoni M (2018). Clinical relevance of genetic variants of gonadotrophins and their receptors in controlled ovarian stimulation: a systematic review and meta-analysis. Hum Reprod Update. **24**(5):599–614. doi:10.1093/humupd/dmy019. |
| Sesh K. Sunkara | Ben-Nagi J, Jones B, Naja R, Amer A, Sunkara S, SenGupta S, Serhal P (2019). Live birth rate is associated with oocyte yield and number of biopsied and suitable blastocysts to transfer in preimplantation genetic testing (PGT) cycles for monogenic disorders and chromosomal structural rearrangements. Eur J Obstet Gynecol Reprod Biol X. **4**:100055. doi:10.1016/j.eurox.2019.100055. |

*Key publications in other years.
