# Supplementary material for: Effect of Genetic Variants of Gonadotropins and Their Receptors on Ovarian Stimulation Outcomes: A Delphi Consensus
Source: Front Endocrinol (Lausanne). 2022 Feb 1;12:797365. doi: 10.3389/fendo.2021.797365 (PMC8844496; doi:10.3389/fendo.2021.797365)
Supplement: Supplementary file 2 [file Table_2.docx]

## *Supplementary Table 2*

| **Statement number** | **Motivations Supporting Disagreement** |
| --- | --- |
| **1** | Although cohort studies demonstrated the need for higher gonadotropin doses in GG patients, meta-analysis of these studies failed to show significant differences in the FSH consumption. |
|  | It depends on the study population. |
|  | We do not have any RCT with an adequate sample size and statistics to prove this conclusion. |
| **2** | It depends on the study population. The studies were carried out in homogeneous populations. |
|  | Information not replicated in every trials. |
| **3** | It depends on the study population. |
|  | Statement based on observational studies based on heterogeneous populations and with heterogeneous protocols of ovarian stimulation. |
|  | Again, this conclusion is based on a limited number of patients in a retrospective design. We need a RCT to prove it... |
|  | Our experience shows No statistically significant difference. |
| **4** | Not supported in the literature. |
|  | It depends on the study population. The studies were carried out in homogeneous populations. |
|  | Information obtained from heterogeneous populations in observational trials, with heterogeneous protocols of ovarian stimulation. |
|  | Only limited retrospective studies or with a small sample size concluded that. In terms of biology this statement is correct, however needs to be proven in an RCT. |
|  | The literature doesn't show this relation. |
|  | Our experience shows no significant statistical difference. |
| **4 (revote*)** | Further studies are needed for this statement. |
|  | Again, the available evidence does not allow such a generalized statement across all ovarian response categories. |
| **5** | Absence of evidence in the literature. |
|  | This finding is only a limited evidence from some retrospective studies, which needs an RCT to be proven. |
|  | Not proven. |
|  | This correlation was detected in specific populations. Further studies are needed to show this correlation. |
| **6** | Data obtained from heterogeneous populations in observational trials. |
|  | This main conclusion is from a Chinese population. To extrapolate we should include more patients from different populations and an RCT design. |
|  | It seems that the Thr allele is not contributing. |
|  | Further studies are needed to accept this, as this finding is not strong. Studies show that comparing Thr/Thr carriers versus Ala/Ala carriers, there is no difference in dose duration of gonadotropin stimulation. |
| **6 (revote)** | Few and not properly designed studies. |
| **7** | Data obtained from heterogeneous study populations and not replicated in every trials. |
|  | Again we need a more robust evidence from RCT with more patients included. |
|  | It seems that the Thr allele is not contributing. |
| **7 (revote)** | Depends very much on how dosage is adjusted. |
|  | The number of subjects included and the design were not adequate. |
|  | The available evidence does not allow such a statement. In fact, that has not been proven across all categories of ovarian response. |
|  | I don’t think has been proven. |
| **8** | Although data is based on 3 studies it seems that there is a considerable effect size with a quite an important difference in gonadotropin consumption. Maybe a softened statement. |
|  | The quoted literature was produced mainly by one group and has not been replicated in studies of sufficient quality. |
|  | It depends on the study population. The studies were carried out in homogeneous populations. The results also need to be evaluated in mixed populations. |
|  | Limited studies with inadequate and retrospective sample sizes. therefore, we cannot conclude this. we can only propose more studies with adequate sample size and design. |
|  | Further studies are needed to confirm these findings in all ethnic groups. |
| **9** | For the same reason as before: all studies from only one group and no independent validation. |
|  | It depends on the study population. The studies were carried out in homogeneous populations. The results also need to be evaluated in mixed populations. |
|  | Limited and retrospective or small number of patients studied. |
|  | Further studies are needed to confirm these findings in all ethnic groups. |
| **8/9 merged (revote)** | A/A allele carriers produce higher number of oocytes than G/G carriers. |
|  | From our personal experiences, literature, have been shown that ethnicity plays a fundamental role in SNPs. Also by definition we need 1% of population in the country to carry the SNP to have clinical application. |
|  | First studies were not correctly planned. |
| **10** | N/A |
| **11** | Not proven. |

*Any statement that did not achieve consensus in Round 2 was discussed and reworded in Round 3, in order to revote with the extended panel. FSH, follicle stimulating hormone; RCT, randomised control trial; SNP, single nucleotide polymorphisms
